# Vitamin D-loaded lipid nanoparticles: antioxidant properties, preparation, optimization, and in vitro characterization

**DOI:** 10.1007/s13346-025-01946-1

**Published:** 2025-08-23

**Authors:** Khadeejeh AL-Smadi, Mohammad Imran, Ayyah Abdoh, David Liu, Khanh Phan, Newton Andreo Filho, Vania Rodrigues Leite-Silva, Yousuf Mohammed

**Affiliations:** 1 Faculty of Health, Medicine and Behavioural Science, Frazer Institute, The University of Queensland, Brisbane, QLD 4102 Australia; 2 School of Pharmacy, The University of Queensland, Brisbane, QLD 4102 Australia; 3 Departamento de Ciências Farmacêuticas, Instituto de Ciências Ambientais, Químicas e Farmacêuticas, Universidade Federal de São Paulo, UNIFESP- Diadema, Brazil

**Keywords:** LNP, TEM, ROS, Vitiligo, Cytotoxicity, Zeta potential

## Abstract

Vitamin D_3_-loaded lipid nanoparticles (Vit D_3_-LNP), integrated into an azulene cream, were developed to enhance the topical delivery and stability of Vitamin D_3_. The LNP was formulated using a lipid mixture and hot homogenization-ultrasonication, with comprehensive characterization revealing a particle size of 153.9 nm, a high zeta potential (-54.3 mV), and a PDI of 0.216, which TEM confirmed. Encapsulation efficiency was high (96.98%), indicating successful incorporation of Vitamin D_3_ within the lipid matrix. Stability studies revealed the impact of light exposure on Vitamin D_3_ degradation. In vitro, release, and skin penetration studies using Franz diffusion cells and two-photon microscopy demonstrated enhanced drug permeation and retention in deeper skin layers with the cream formulation. Cell Viability test confirmed high cell viability (~ 80–100%) for both free Vitamin D_3_ and the LNP formulation; also inflammation test showed a significant reduction in ROS levels with Vitamin D_3_-LNP treatment. These findings highlight the therapeutic value of LNP in managing conditions like Vitiligo, providing insights into the design of stable, effective Vitamin D_3_ delivery systems for dermal applications, and offering a promising approach for advanced skin treatments.

**Graphical Abstract:**

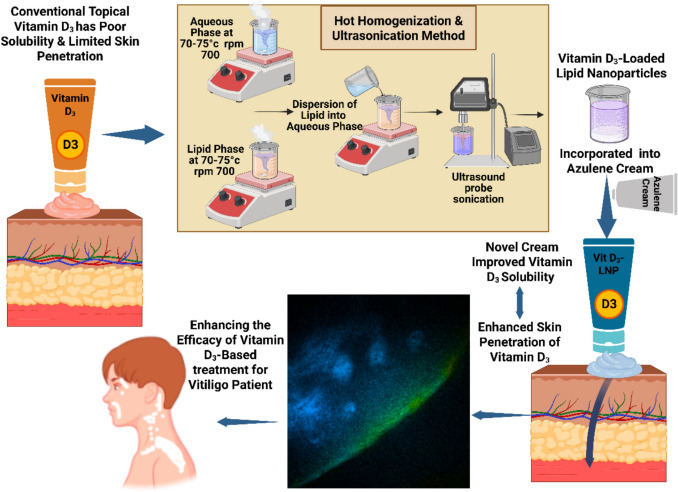

## Introduction

Vitamin D deficiency has become a prevalent health concern worldwide, with implications ranging from bone disorders to immune system dysregulation. While oral supplementation remains a common approach for addressing this deficiency, topical application offers a promising alternative, particularly in dermatological conditions such as vitiligo. Vitiligo, characterized by the loss of skin pigmentation, presents a unique challenge as conventional treatments often fall short of achieving satisfactory repigmentation. Topical Vitamin D application has shown potential in promoting melanocyte proliferation and differentiation, offering a targeted therapeutic strategy for Vitiligo management [1]. To enhance the efficacy of such topical treatments, additional agents with complementary properties may be incorporated. Azulene (sodium 1,4-dimethyl-7-isopropylazulene-3-sulfonate), known for its anti-inflammatory and skin-soothing effects, represents one such candidate. Derived from common yarrow, chamomile, and absinthe, it is a compound found in cosmetic products and has demonstrated potential in managing skin inflammation. In the context of vitiligo, its anti-inflammatory properties may support skin regeneration and reduce local oxidative stress, thereby complementing the action of Vitamin D. In Italy, it is available for topical use in a colloidal vehicle under the brand name Veralga azulene^®^ gel [2].

The possibility of delivering vitamin D through the skin either to improve skin conditions or to reach internal organs represents the primary target. The inability of highly lipophilic active substances, such as Vitamin D, to pass through the stratum corneum barrier represents a challenging task in administering these compounds topically. Additionally, when administered in cream formulations, Vitamin D tends to deposit on the skin surface due to its affinity with the vehicle. To overcome this limitation and to promote Vitamin D permeation through the skin, despite its physicochemical characteristics, specialized transdermal delivery systems with penetration enhancement properties are required [3, 4].

Nanotechnology-based drug delivery systems have gained widespread attention for their ability to enhance the therapeutic efficacy of various drugs. Among them, nanoparticles, which are solid colloidal particles typically ranging in diameter from 1 to 100 nm, are widely explored due to their unique physicochemical properties. These include metal nanoparticles, polymeric micelles, polymeric nanoparticles, and lipid-based systems such as liposomes and solid lipid nanoparticles. Their capacity to improve drug stability, bioavailability, and targeted delivery makes them suitable not only for pharmaceuticals but also for applications in cosmetics and skincare [5–7]. Lipid nanoparticles (LNPs), introduced in the 1990 s, have emerged as particularly promising carriers in the field of nanomedicine. Composed of biocompatible and biodegradable lipids, LNPs encapsulate active pharmaceutical ingredients (APIs) and protect them from degradation processes such as oxidation [8]. These nanoparticles exhibit minimal skin irritation, high drug-loading capacity, and the ability to provide controlled and sustained release [9]. When applied topically, they form an occlusive film that enhances skin permeation and facilitates drug retention in the targeted skin layers, increasing therapeutic effectiveness [10, 11].

Several studies have indicated that formulations containing the same drug at the same concentration can exhibit varying drug delivery rates through the skin due to the physicochemical properties of the vehicle used. Moreover, formulations employing the same ingredients can also result in different drug delivery rates based on the droplet size of the emulsion. In vitro drug release tests provide valuable insights into evaluating the impact of formulation factors such as dose formulas, vehicle composition, and drug solubility in vehicles. These considerations are crucial when formulating topical preparations [12, 13].

Formulation components have two primary effects on skin permeation. Firstly, they can modify the lamellar structure of intercellular lipids in the stratum corneum. These components can potentially enhance or impede the permeation of the drug through the skin by altering the organization and packing of the intercellular lipids. This, in turn, influences the pathway and rate at which drug molecules can traverse the stratum corneum. Secondly, formulation components can impact the solubility properties of lipids. The solubility of both the drug and the vehicle in these lipids can affect drug permeation through the skin. The lipid composition of the stratum corneum is a significant determinant of skin permeability. The composition of lipids in the stratum corneum plays a vital role in determining skin permeability. Formulation components can modulate the solubility of the drug in these lipids, consequently influencing the drug’s ability to penetrate the skin [14–16].

The size of LNP plays a crucial role in the stability of the formulation, drug release rate, and its behavior in the body. Various factors influence particle size, including lipid and surfactant properties, production techniques, and processing conditions (such as time, temperature, pressure, and the number of cycles). In both high-pressure homogenization and high-shear homogenization, larger particle sizes are observed with higher melting point lipids, attributed to increased viscosity of the dispersed phase [17]. The lipid composition significantly impacts the quality of LNP dispersion, and variations in lipid composition from different suppliers and batches are noteworthy. A higher lipid content (> 5–10%) increases particle size and polydispersity due to enhanced viscosity, which affects homogenization efficiency and particle agglomeration. The surfactant’s properties and concentration also influence the size and efficacy of LNP. Smaller particle sizes result in an increased surface area, leading to thermodynamic instability and phase separation through Ostwald ripening. Adequate surfactant concentration is crucial to cover newly formed surfaces and prevent phase separation by reducing interfacial tension [18], which, in turn, can impact the overall efficacy of the LNP formulation. Surfactant/cosurfactant mixtures have been observed to enhance LNP stability and reduce particle size compared to formulations with surfactant alone. The type of surfactant used influences the homogenization parameter required for a specific formulation. For instance, LNP dispersions stabilized with ionic surfactants exhibit smaller particle sizes compared to those stabilized by nonanoic surfactants. Researchers emphasize the need to determine the optimum surfactant concentration for each formulation to ensure stability and efficacy [19].

We have undertaken the present study to develop a topical Vitamin D formulation optimized with lipid nanoparticles (LNPs) to enhance its effectiveness in treating skin-specific conditions, such as Vitiligo. Unlike oral supplementation, which is subject to systemic metabolism and distribution, a localized topical approach offers more targeted therapeutic action. Additionally, to address the limitations related to skin penetration and surface deposition of Vitamin D, the formulation utilizes the unique properties of LNPs, including enhanced penetration, physicochemical stability, and controlled release, to maximize its therapeutic potential in dermatological applications.

## Materials and methods 

### Materials

Sigma-Aldrich, Australia, provided standard Vitamin D_3_ (Purity ≥ 99.0%, HPLC grade), Glyceryl Monostearate (Kolliwax^®^ GMS II), and Polyvinylpyrrolidone (PVP) (Kollidon^®^ 30). PEG-35 Castor Oil (CO) and Flaxseed Oil (FSO) were also obtained from Sigma-Aldrich, Australia. Propylene Glycol Monolaurate (Lauroglycol FCC) (PML) and Tefose^®^ 1500 (PRG-6 Stearate/PEG-32 Stearate) (PEG) were gifted by Gattefosse Pharmaceuticals, Australia. Ethoxylated Oleyl Alcohol 20 OE (EOA – Chemonic OE20) was sourced from Croda Pharma.

Our lab sourced HPLC-grade acetonitrile (ACN), methanol, medium-chain triglycerides (Caprylic/Capric Triglyceride, CCT), ultra-pure water (Milli-Q, IONPURE, USA), Propylene Glycol (PG), Disodium EDTA, Poloxamer 407, Shea Butter Cetyl Esters (AGREEN W), Glyceryl Stearate, Cetearyl Alcohol (combined with Polysorbate 60), and Phenoxyethanol***.***

### Preparation of Vitamin D_3_-loaded lipid nanoparticles

#### Development of an HPLC method for accurate quantification of Vitamin D_3_

The Shimadzu HPLC system was utilized to quantify Vitamin D_3_. The setup included a UV–vis detector, column oven, autosampler, pump module, and a Shimadzu Shim-pack VP-ODS C18 column (5 µm, 4.6 mm ID × 250 mm L), all from Shimadzu, Japan. Standard and Working Solution Preparation: To create a 100 µg/mL Vit-D_3_ stock solution, 1 mg of Vitamin D_3_ standard chemical was dissolved in 1 mL of the mobile phase solvent of acetonitrile: methanol (60:40). Vitamin D_3_ working standard solutions, ranging from 0.25 µg/mL to 10 µg/mL (0.25,0.5,1,2.5,5,8 and 10), was prepared through serial dilution for the establishment of linearity in calibration graphs [20]. The calibration curve, constructed with known concentrations of Vitamin D_3_ standard, exhibits excellent linearity over a specified concentration range, ensuring a reliable quantitative assessment using the HPLC method. Chromatographic Conditions: The chromatographic separation employed a mobile phase of Acetonitrile: Methanol (60:40) at a flow rate of 1.0 mL/min. A 10 µL injection volume was used for each single analysis. The separation occurred on a C18 column with the oven temperature set at 30°C. Detection was performed at a wavelength of 265nm using a simple UV–vis detector. Vitamin D_3_ was identified by comparing its responsive peaks with the standards, ensuring identical retention times.

### Saturated solubility of Vitamin D_3_ in liquid lipids

The saturated solubility of Vitamin D_3_ was evaluated in four selected liquid lipids—Caprylic/Capric Triglyceride (CCT), Castor Oil (CO), Flaxseed Oil (FSO), and Propylene Glycol Monocaprylate (PML)—under controlled conditions to support formulation development. An excess amount of Vitamin D_3_ was incrementally added to each lipid at 5 °C to minimize degradation and thermal instability. The dispersions were agitated on a shaker for 48 h to ensure equilibrium. After incubation, the samples were centrifuged at 5000 rpm for 15 min, and the supernatants were then filtered through a 0.45 µm membrane filter. The filtrates were then diluted 100-fold in methanol and analyzed using the validated HPLC method.

### Compatibility test of solid and liquid lipids

To assess the compatibility between the solid and liquid lipids, a Laser Confocal Microscope Olympus LEXT OLS5100 was employed. A mixture of the two lipids was prepared in a 1:1 ratio, as per the intended formulation. The mixture was then heated slightly above the melting point of the solid lipid to ensure complete melting and facilitate proper mixing. Upon heating, the lipid blend was carefully observed for any signs of phase separation, precipitation, or the presence of visible particles. Once a clear and homogeneous solution was obtained, a small drop of the mixture was placed on a clean glass microscope slide and covered with a coverslip. The prepared slide was then examined under an optical microscope using both transmitted and polarized light to enhance visualization of any crystalline structures or phase discontinuities. The lipid mixture appeared uniform and well-dispersed, with no evidence of phase separation, crystal formation, or oil droplets observed under microscopy. These observations indicated good compatibility between the solid and liquid lipids, suggesting that each component was thoroughly and evenly dispersed within the other.

### Nanoparticle synthesis through hot homogenization and ultrasonication

Lipid Nanoparticles (LNPs) can be produced using various techniques, one important strategy is the use of Hot Homogenization and Ultrasonication.

#### Hot homogenization and ultrasonication

**Fig. 1:**
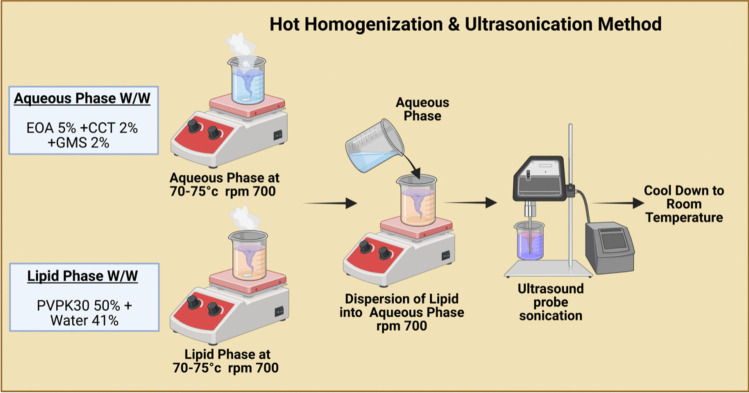
Hot Homogenization and Ultrasonication Method. Created with Biorender.com

Lipid nanoparticles (LNPs) were prepared using a hot homogenization and ultrasonication method (Fig. 1). The lipid phase, consisting of Caprylic/Capric Triglyceride (CCT), Glyceryl Monostearate (GMS), and Ethoxylated Oleyl Alcohol 20 OE (EOA), was heated to 70–75°C until it was fully melted. Separately, the aqueous phase containing Polyvinylpyrrolidone K30 (PVP K30) and water was also heated to the same temperature. The aqueous phase was gradually added to the lipid phase under continuous stirring using a high-shear homogenizer. The mixture was homogenized and then subjected to ultrasonication using a probe sonicator to reduce the particle size and enhance the overall homogeneity of the dispersion [21].

Schematic representation of the Hot Homogenization and Ultrasonication Method for the preparation of lipid nanoparticles. The process involves heating and mixing of aqueous (Polyvinylpyrrolidone (PVP) (Kollidon^®^ 30) + water) and oil (Ethoxylated Oleyl Alcohol 20 OE (EOA – Chemonic OE20), Caprylic/Capric Triglyceride (CCT), and Glyceryl Monostearate (Kolliwax^®^ GMS II)) phases at 70–75 °C and rpm 700, followed by emulsification under stirring and size reduction using tip ultrasonication.

### Optimization of formulation parameters

The development of the lipid nanoparticle dispersion employed the Hot Homogenization and Ultrasonication method, utilizing an initial formulation that demonstrated promising results. The outcomes of this formulation were encouraging, as determined through collaborative laboratory assessments. Formulation consisted (for 100 g formulation) of 5% Ethoxylated Oleyl Alcohol 20 OE (EOA – Chemonic OE20), 2% Medium Chain Triglycerides (Caprylic/Capric Triglyceride), 2% Glyceryl Monostearate (GMS – Kolliwax GMS II), and 50% Polyvinyl Pyrrolidone k30 aqueous dispersion (PVPk30 – Kollidon 30), prepared by mixing 2 g of PVPk30 with 98 g of water to achieve a 2% concentration. The remaining 41% of the formulation was composed of water. For emulsification, both the oil and aqueous phases were heated to approximately 70–75 °C, followed by the slow addition of the aqueous phase into the oil phase under stirring. Subsequently, the mixture was removed from the heat and stirred until it cooled to approximately 30 °C. Finally, to generate nanoparticles, ultrasound processing was used with 10 cycles of 30 s on and 30 s off, and the ultrasound amplitude was set at 40%. For this formulation, further optimization of both the process parameters and formulation composition was necessary to achieve the desired particle size and ensure long-term stability of the dispersion.

#### Process development for lipid nanoparticles loaded with Vitamin D_3_

Once the lipid nanoparticle dispersion yielded a partially monodisperse system with a z-average size ranging from 100 to 400 nm, further optimization of the process and formulation was carried out to enhance nanoparticle properties. This optimization focused on refining critical parameters during both the emulsification and ultrasonication steps. During emulsification, the mixture was heated to at least 10 °C above the melting point of the solid lipid. Following the addition of the aqueous phase, the temperature was maintained at 70–75°C for a minimum of 10 min. Stirring was performed at varying speeds—400, 700, and 1000 rpm—to evaluate its impact on emulsion quality. All emulsification steps were conducted using glass containers to ensure effective transmission of ultrasound waves. For the ultrasonication process, a probe-type sonicator was employed with intermittent cycles (30 s on/30 s off) applied for 5, 10, or 15 min at different amplitudes (30%, 40%, and 50%), as outlined in the experimental design (Table 1).

**Table 1:** Process Development. Experimental design matrix for process optimization of Vitamin D_3_-loaded lipid nanoparticle formulation using a Box–Behnken Design. The three independent variables—stirring speed during emulsification (rpm), ultrasound processing time (min), and ultrasound amplitude (%)—were evaluated at three levels. The design comprises 15 randomized runs, including 3 center points to assess process reproducibility and statistical robustness. The orange column indicates the sequence in which the experiments should be performed

Std Order	Run Order	Pt Type	Blocks	Stirring (rpm)	Ultrasound time (min)	Ultrasound amplitude (%)
14	1	0	1	700	10	40
2	2	2	1	1000	5	40
1	3	2	1	400	5	40
6	4	2	1	1000	10	30
9	5	2	1	700	5	30
13	6	0	1	700	10	40
3	7	2	1	400	15	40
8	8	2	1	1000	10	50
10	9	2	1	700	15	30
15	10	0	1	700	10	40
5	11	2	1	400	10	30
11	12	2	1	700	5	50
4	13	2	1	1000	15	40
12	14	2	1	700	15	50
7	15	2	1	400	10	50

#### Formulation development for lipid nanoparticles loaded with Vitamin D_3_

In the formulation development for lipid nanoparticles loaded with Vitamin D_3_, several parameters were optimized to achieve the desired nanoparticle characteristics. These parameters included the amount of surfactant, the proportion of ionic and non-ionic surfactants, the types of surfactants, the ratio of aqueous to oil phases, and the balance between solid and liquid lipids. Specifically, the impact of different surfactant concentrations (3%, 5%, and 7%) was evaluated, along with various ratios of ionic (Sodium Lauryl Sulfate) and non-ionic surfactants such as Ethoxylated Oleyl Alcohol (EOA), Kolliphor EL (ECO), and Tween 20 (TW), to achieve optimal charge and stability. The effect of different proportions of aqueous to oil phases (2%, 4%, and 6%) and varying ratios of solid to liquid lipids (5:5, 6.5:3.5, and 8:2) was also assessed.

To optimize these factors, a Central Composite Design (CCD) with three variables—surfactant concentration, oil phase concentration, and surfactant type—was implemented, and 27 experiments were conducted, including center points. The formulations were evaluated for Z-average, polydispersity index (PDI), Zeta Potential, and stability at various time points (0, 1, 7, and 15 days). Minitab software was used to analyze the data and identify the best formulation. The design consisted of 27 randomized runs, each representing a specific combination of surfactant concentration, oil phase concentration, and surfactant type. The experiments were carried out according to the run order indicated in the orange column of (Table 2).

**Table 2:** Formulation Development. Experimental design matrix for formulation optimization of Vitamin D_3_-loaded lipid nanoparticles using a Central Composite Design (CCD). Three formulation variables—surfactant concentration (%), oil phase concentration (%), and surfactant type (categorical: Ethoxylated Oleyl Alcohol (EOA), Kolliphor EL (ECO), and Tween 20 (TW)—were investigated to evaluate their influence on particle size (Z-average), polydispersity index (PDI), and zeta potential. The design includes 27 randomized runs comprising factorial, axial, and center points to support robust statistical modeling and optimization. The orange column indicates the sequence in which the experiments should be performed

Std Order	Run Order	Pt Type	Blocks	[surfactant] %	[Oil phase] %	Surfactant type
6	1	−1	1	7.83	4.00	EOA
17	2	−1	1	5.00	6.83	ECO
11	3	1	1	7.00	2.00	ECO
20	4	1	1	7.00	2.00	TW
12	5	1	1	3.00	6.00	ECO
1	6	1	1	3.00	2.00	EOA
8	7	−1	1	5.00	6.83	EOA
19	8	1	1	3.00	2.00	TW
16	9	−1	1	5.00	1.17	ECO
15	10	−1	1	7.83	4.00	ECO
24	11	−1	1	7.83	4.00	TW
13	12	1	1	7.00	6.00	ECO
5	13	−1	1	2.17	4.00	EOA
21	14	1	1	3.00	6.00	TW
14	15	−1	1	2.17	4.00	ECO
2	16	1	1	7.00	2.00	EOA

By systematically varying key process parameters and evaluating the resulting nanoparticle properties, the formulation and processing conditions for Vitamin D_3_-loaded lipid nanoparticles were optimized to enhance stability, uniformity, and potential drug delivery efficacy.

### Development of a cream vehicle for the lipid nanoparticles

To enable the topical application of lipid nanoparticles, a suitable cream vehicle was developed with emulsifying and moisturizing components. In the initial phase (Phase A)(Table 3), Poloxamer was incorporated into water and stirred at 400 rpm for 5 min using an overhead stirrer until complete dissolution, with the option of applying heat if necessary. Subsequently, Propylene Glycol (PG) and EDTA were introduced, and the mixture was stirred at 400 rpm until homogeneity was achieved within a 5-min timeframe. Simultaneously, in Phase B, Glyceryl Stearate, Cetearyl Alcohol (and) Polysorbate 60, Caprylic/Capric Triglyceride, and Shea Butter Cetyl Esters were weighed and placed in a 50 mL beaker, then subjected to heating using a water bath. Integrating Phase A and B involved withdrawing both phases from the water bath when the temperature reached no more than 75°C. Phase B was gradually added to Phase A, and homogenization was conducted at 6600 rpm for 15 min, starting at 3200 rpm and increasing progressively to the target speed before timing commenced. After homogenization, the speed was reduced back to 3200 rpm. Then, 0.03% w/w Azulene (30 mg per 100 g of cream) was added and mixed thoroughly to ensure uniform distribution throughout the formulation [2]. Next, Phenoxyethanol was incorporated into the mixture. Continued stirring at 3200 rpm for an additional 5 min. Finally, to remove any entrapped air bubbles, sonication was applied for 7 min.

**Table 3:** Cream Formulation Materials. Composition of the base cream formulation used for incorporation of Vitamin D_3_-loaded lipid nanoparticles. The cream was prepared in two phases: Phase A includes aqueous components: Water, Propylene Glycol (PG), EDTA, and Poloxamer 407, while Phase B contains the lipidic and emulsifying agents including Shea Butter Cetyl Esters, Caprylic/Capric Triglyceride, Glyceryl Stearate, and Cetearyl Alcohol (and) Polysorbate 60. Azulene (0.03% w/w) and Phenoxyethanol (0.8% w/w) were incorporated post-emulsification

PHASE	MATERIALS	%w/w
A	Water	79.15
	Propylene glycol (PG)	3.0
	EDTA	0.05
	Poloxamer 407	1.0
B	Shea Butter Cetyl Esters	3.0
	Caprylic/Capric Triglyceride	5.0
	Glyceryl Stearate	3.0
	Cetearyl Alc. (and) Polysorb.60	5
	Phenoxyethanol	0.8
	TOTAL	100

To incorporate the Vitamin D_3_-loaded lipid nanoparticles (LNPs) into the cream, the base formulation was first prepared using 70% w/w water instead of 80% w/w, resulting in approximately 90 g of cream after emulsification and cooling to 35–40 ºC. This adjustment was made to leave a 10 g space for the addition of the LNP dispersion. A total of 10 g of the nanoparticle dispersion, containing 100 µg/g of Vitamin D_3_ (equivalent to 1 mg of Vitamin D_3_), was gradually added to the cream under gentle stirring at the lowest possible RPM to ensure homogeneous distribution. The addition was carried out when the cream temperature was below 35 ºC to prevent nanoparticle destabilization. Shear was also minimized to avoid incorporation of air during mixing, resulting in a final cream weight of 100 g with a Vitamin D_3_ concentration of 0.01% w/w.

### Characterization of LNP loaded with Vitamin D_3_

#### Physical characterization using the DLS technique (Particle Size, PDI, and Surface Charge) and TEM

##### Dynamic Light Scattering (DLS) and zeta potential analysis

The physical characterization of Vitamin D_3_-loaded lipid nanoparticles (LNPs) was conducted using a Zetasizer (Malvern Zetasizer Nano Instruments, UK). For particle size and polydispersity index (PDI) analysis, the LNP aqueous dispersion was diluted 100 times with Milli-Q^®^ water. Zeta potential was measured after diluting 50 µL of the sample in 10 mL of Milli-Q^®^ water, with conductivity adjusted to 50 mS/cm using a 0.9% w/v sodium chloride solution. All measurements were performed in triplicate at 25 ± 2 °C [22].

##### Transmission Electron Microscopy (TEM)

The morphology of optimized Vitamin D_3_-loaded LNPs was analyzed using a high-resolution transmission electron microscope (TEM) (TECHNAI-G2, 200 kV, HR-TEM, Netherlands) after 50-fold dilution with Milli-Q^®^ water. A drop of the sample was placed on a carbon-coated grid, adhered for 1 min, and negatively stained using 2% uranyl acetate (1 min) followed by 0.5% lead citrate (3 min) under CO₂-controlled conditions. Grids were rinsed with double-Milli-Q^®^ water, dried, and stored in a protective case. Imaging was conducted in high-resolution mode to observe particle shape, distribution, and aggregation [22–24].

### Thermogravimetric characterisation of LNP loaded with Vitamin D_3_

Differential Scanning Calorimetry (DSC) was employed to evaluate the thermal properties of lipid nanoparticles (LNPs) and Vitamin D_3_ -loaded LNPs. Before analysis, samples were freeze-dried using a VirTis Freeze Dryer to eliminate water and solvents that could interfere with thermal measurements. For freeze-drying, 2 mL of each formulation was transferred into sterile, compatible tubes and pre-frozen at −80 °C for at least 24 h. The frozen samples were then placed on the freeze-dryer shelf, and the primary drying was conducted at −40 °C under a vacuum of 50–100 millitorr for 12–24 h. Once drying was complete, tubes were sealed tightly to prevent moisture reabsorption. After lyophilization, the dried samples were weighed to ensure 5–10 mg was available for thermal analysis [25, 26].

Thermal analysis was performed using a DSC (PerkinElmer Corp., USA) under a nitrogen atmosphere to identify thermal transitions, including melting, crystallization, and oxidation. Approximately 10 mg of the dried sample was placed in an aluminum pan, with an empty pan serving as the reference. The temperature program ranged from 30 °C to 600 °C at a heating rate of 10 °C/min, allowing precise monitoring of phase changes. Special attention was given to transitions occurring between 32 °C and 37 °C, which are relevant for skin-contact applications. This setup ensured accurate characterization of the nanoparticles and the encapsulated Vitamin D_3_ by eliminating moisture interference and providing consistent thermal conditions [27].

### Determination of encapsulation efficiency

The LNPs, including both plain LNP and drug-loaded LNP (DLPL), were prepared to determine the total amount of Vitamin D_3_. A known volume of the nanoparticle suspension was mixed with an equal volume of methanol and vortexed thoroughly for a few minutes to ensure the complete dissolution of the lipids and the release of Vitamin D_3_. For free Vitamin D_3,_ another aliquot of the nanoparticle suspension was centrifuged at 3000 rpm for 30 min using an Amicon^®^ Ultra-15 Centrifugal Filter tube to separate unentrapped Vitamin D_3_. Both samples were analyzed using a suitable analytical method, such as high-performance liquid chromatography (HPLC), to quantify the free and total amount of Vitamin D_3_ in LNP [28–30].

The encapsulation efficiency (EE) is calculated using the formula in Eq. (1):

1 $$\mathrm{EE}\left(\%\right)=\frac{Total\;amount\;of\;Vitamin\;D_3\;in\;LNP-amount\;of\;free\;Vitamin\;D_3\;in\;LNP}{Total\;amount\;of\;Vitamin\;D_3\;in\;LNP}\times100\%$$

#### Chemical stability of Vitamin D_3_ in LNP

##### Thermal and light stability test

The _thermal_stability of Vitamin D_3_ loaded in lipid nanoparticles was evaluated by subjecting aliquots of the nanoparticle dispersion to various temperature conditions. These conditions included elevated temperatures of 40 °C, room temperature, freezing (−20 °C), as well as fridge temperatures of 4 °C. The samples were stored in tightly sealed containers and maintained at the specified temperatures for a predetermined duration of 30 days. After incubation, the stability of Vitamin D_3_ was assessed using the high-performance liquid chromatography (HPLC) technique. Any changes observed compared to initial measurements were interpreted as temperature-induced degradation susceptibility of Vitamin D_3_ (thermal instability) [31]. To investigate the light stability of Vitamin D_3_, one sample of the nanoparticle dispersion loaded with Vitamin D_3_ was transferred to a light-resistant container. This container was exposed to daylight (normal room light) in a transparent, tightly sealed container, while the control sample was shielded from light to serve as a reference [32]. Over a specified exposure period of 30 days, the samples were monitored for changes in Vitamin D_3_ content. Analysis techniques such as high-performance liquid chromatography (HPLC) were used to assess Vitamin D_3_ degradation. Comparison with the control sample was used to determine the impact of light exposure on the stability of Vitamin D_3_ in the lipid nanoparticles.

### Characterization of cream loaded with LNP

To determine the flow behavior of the formulations, a steady-state shear stress sweep was conducted using an Anton Paar rheometer equipped with a 40 mm parallel plate geometry and a Peltier temperature-controlled stage set at 32 °C. To minimize slippage artifacts, 180-grit sandpaper was affixed to both the upper and lower measurement surfaces, enhancing mechanical interlocking with the sample. Samples (both the base cream and the LNP-loaded cream) were carefully loaded onto the Peltier plate, and the upper geometry was adjusted to a standardized measurement gap of 500 µm. Excess material was trimmed to ensure a uniform loading profile. Each sample underwent pre-conditioning via low-shear pre-shearing at 1 s⁻^1^ for 60 s to erase any shear history, followed by a 120-s rest period to allow for structural recovery, thereby simulating application and recovery dynamics. [33, 34]. A logarithmic shear stress ramp from 1 to 350 Pa was applied to capture the formulation’s viscosity profile as a function of shear, enabling quantification of shear-thinning (pseudoplastic) behavior, commonly observed in semisolid dermatological systems. The rheological profiles of both the base cream and LNP-loaded cream were compared to assess any impact of nanoparticle incorporation on flow behavior. This rheological approach aligns with Quality by Design (QbD) principles to evaluate critical quality attributes (CQAs), particularly spreadability and in-use performance [35–37]. Yield stress, defined operationally as the minimum applied stress required to initiate irreversible deformation, was derived from the point of inflection on the viscosity-shear stress curve. This transition marks the shift from elastic (solid-like) to viscous (flowable) behavior. Yield stress is considered a key performance indicator for semisolid topical systems, as it correlates with spreadability during application and physical stability during storage, aligning with criteria emphasized in QTPP development frameworks. All rheological measurements were performed in triplicate to ensure data robustness and reproducibility [33, 34, 38].

Texture analysis was conducted using a TA.XTplus Texture Analyzer (Stable Micro Systems) equipped with a TA10 cylindrical probe and a 5000 g load cell in compression mode, simulating the tactile forces encountered during topical application [39, 40]. The test employed a double compression cycle (two-cycle stress-relaxation test) to evaluate the mechanical response of the cream under repeated deformation, replicating typical consumer interaction [41, 42]. The probe descended at a constant test speed of 1.00 mm/s until a trigger force of 3.00 g was detected, after which it continued to a defined penetration depth of 5.00 mm [43]. The instrument then returned to the starting position, with a 5-s pause before initiating the second compression. Data acquisition was set to a high temporal resolution (100 Hz during compression and 20 Hz during the return stroke) to capture transient force responses accurately. Testing was performed at ambient temperature using freestanding block samples (10.86 mm in length) without fixture constraint, allowing for unimpeded deformation [42, 44].

From the force-distance curves, key texture attributes were quantitatively evaluated. Hardness was defined as the peak force during the first compression, reflecting the formulation’s firmness. Cohesiveness, calculated as the ratio of areas under the second and first compressions, indicated structural retention upon repeated stress. Adhesiveness represented the work required for probe detachment, while elasticity (springiness) described the formulation’s ability to recover its original shape after deformation. These parameters were compared between the base cream and the LNP-loaded formulation to determine the effect of nanoparticle incorporation on mechanical behavior and user experience [34, 36, 43].

### In vitro skin penetration test

#### Human skin preparation

Full-thickness human skin samples were obtained from patients undergoing abdominoplasty at Brisbane (QLD) hospitals following elective surgery, with ethical approval granted by the University of Queensland Human Research Ethics Committee (2018/HE001721). The full-thickness skin was cleaned by separating the underlying fat and stored at −20 °C until use. Before mounting the skin, a piece of female frozen full-thickness abdominal skin, age 49 years, was thawed slowly, and the epidermal layer was separated using a heat separation protocol [45]. The heat-soaking technique for preparing epidermal sheets was adapted from a method originally developed by Christophers and Kilgman in 1963 [46]. To prepare the skin samples, the tissue was placed into a 1000 mL beaker of water heated to 60 °C for 60 s. After heat treatment, the tissue was laid dermis-side-down on a ceramic plate covered with a paper towel. Using a scalpel, an initial incision was made through the epidermis and partially into the dermis at one corner of the sample to begin the separation process. The epidermis was gripped with blunt forceps and slowly peeled away from the dermis, using another set of forceps to stabilize the dermis. Once the epidermis was isolated, it was transferred to cold, deionized water in a glass evaporating basin, ensuring it floated with the stratum corneum side facing up due to its hydrophobic properties, which contrasted with the hydrophilic properties of the viable epidermis. The floating epidermal sheet was carefully transferred onto aluminum foil. The foil was then suspended from a bench with adhesive tape, and the sample was allowed to dry for about one hour. Any remaining water droplets were gently swabbed away with a paper towel. Once dry, the foil containing the epidermal sheet was wrapped in an additional layer of aluminum foil and stored in a freezer at −20 °C.

#### In vitro penetration test (IVPT) using heat-separated human epidermal membranes

The skin permeability of Vitamin D_3_ loaded in lipid nanoparticles (LNP) was evaluated in vitro using a flow-through diffusion system. Human epidermal membranes containing the stratum corneum and viable epidermis were obtained by heat separation and subsequently stored at –20 °C until use. On the day of the experiment, these frozen, heat-separated epidermal membranes were thawed and used as the barrier model. Skin discs (25 mm in diameter) were excised and mounted on vertical Franz diffusion cells with a diffusion area of 1.13 cm^2^. The stratum corneum side was oriented facing the donor compartment, and the membrane edges were sealed with high vacuum grease to prevent leakage. The donor compartment was firmly affixed and sealed using parafilm and blue tags. Test formulations included Vitamin D_3_-loaded lipid nanoparticles (0.01% w/w) in a cream with Azulene (0.03% w/w), as well as control solutions of Vitamin D_3_ (0.01% w/w) and Azulene (0.03% w/w) [47]. A 20 mg sample of each formulation was dispersed in 2 mL phosphate-buffered saline (PBS) and applied to the donor compartment. The receptor compartment (3.2 mL volume) was filled with PBS (pH 7.4) containing 0.05% sodium azide and 0.5% Tween 20 [48]. Franz cells were maintained at 37 °C with a 30-min equilibration period. Receptor phase samples (100 µL) were collected at 0, 2, 4, 6, and 24 h. After each collection, an equal volume of fresh buffer containing the same additives was added to maintain sink conditions and constant volume. Vitamin D_3_ content was quantified using HPLC following centrifugation through Amicon tubes at 10,000 rpm [49, 50]. Drug permeation was evaluated based on the cumulative amount permeated over time. The stratum corneum drug content was determined using 20 tape strips, followed by drug extraction and HPLC analysis. Residual drug in the remaining skin and donor compartment was soaked overnight at room temperature to extract the drug. The extracts were then centrifuged at 10,000 rpm, and the supernatant was analyzed by HPLC. A complete mass balance was calculated by summing drug content across all compartments, with acceptable recovery defined as 80–120%. Each formulation was tested using three different skin donors, with three replicates per donor (*n* = 9). Untreated skin was included as a negative control [51].

### Two-photon penetration studies for nanoparticles loaded with Vitamin D and a dye (Rhodamine B)

The Multiphoton microscopy (MPM) imaging protocol for ex vivo skin, using Rhodamine B-labelled nanoparticles and a cream vehicle containing Azulene, began with sample preparation. Rhodamine B-labelled nanoparticles were prepared with a concentration of 0.5% w/w relative to the lipids by incorporating the dye into the lipid phase before combining it with the aqueous phase. To remove unbound dye, the dispersion was purified using Amicon^®^ ultrafiltration (MWCO 10 kDa), followed by multiple washing steps with deionized water. The filtrates were analyzed to confirm the absence of free Rhodamine B, ensuring that the fluorescence observed in subsequent imaging studies was due to nanoparticle-associated dye. For the experiment, 20 mg of each sample (Rhodamine B-labelled nanoparticles and cream with Azulene) was applied to frozen skin sections with a diffusion area of 1.13 cm^2^. The treated skin samples were incubated, and images were captured at 0, 4, and 24 h to assess permeation across the skin layers. The skin sample was mounted on a glass slide, with 20–80 µL of water applied to ensure hydration, then covered with a glass coverslip secured with tape. A drop of microscope oil was added near the imaging area, and the sample was transferred to the microscope stage, ensuring proper contact between the objective and the oil on the coverslip. Imaging was performed using the DermaInspect^®^ multiphoton tomography system with a Plan-Neofluar 40X/1.30 oil-immersion objective lens, utilizing a titanium-sapphire laser (740 nm excitation wavelength for autofluorescence imaging for Azulene, 840 nm for Rhodamine B, and 770 nm for the combination) [52, 53]. The optical power and exposure time were adjusted based on the skin’s characteristics, starting with an optical power of 23 mW and a 47-s exposure for a 512 × 512 pixel image [54].

Multiphoton microscopy (MPM) and Fluorescence Lifetime Imaging Microscopy (FLIM) images were acquired using the same multiphoton imaging system, with FLIM measurements obtained through time-resolved detection to assess fluorescence lifetime, and MPM used for intensity-based structural visualization. The FLIM measurements were taken using a 350–650 nm bandpass filter to capture emitted light, with 430–450 nm filters for Azulene and 560–580 nm filters for Rhodamine B. A 690-nm shortpass filter was applied to avoid an excitation background. The SPC-830 detector system was used for FLIM analysis, and images of the stratum granulosum and stratum spinosum layers were acquired based on keratinocyte morphology. FLIM images were convoluted with the instrument response function, and decay curves were fitted to each pixel, with color-coded images generated for each decay parameter to visualize the distribution of Rhodamine B and Azulene fluorescence in the skin [52, 53, 55].

### In vitro pro-inflammatory and cell viability tests of LNP

#### Cell viability test of LNP

HaCaT cells were seeded into a 96-well plate at a density of 1 × 10^4^ cells per well in RPMI medium supplemented with 10% fetal bovine serum (FBS), 2 mM glutamine, 100 U/mL penicillin, and 100 µg/mL streptomycin. The plate was incubated at 37 °C with 5% CO₂ for 24 h to allow cell adhesion and reach 70–80% confluency. After this growth phase, the cells were treated with various concentrations (1, 10, 100 µg/mL) of free Vitamin D_3_ and Vitamin D_3_-loaded LNP (Vit D_3_-LNP) and incubated for an additional 24 h. Following treatment, the media in each well were replaced with 200 µL of fresh RPMI medium, and 20 µL of resazurin solution in PBS was added to each well. The plate was incubated for another 1 to 4 h at 37 °C. Finally, absorbance was measured at 570 nm after shaking the plate for 15 min using a microplate ELISA reader (ELX 800, Biotek, USA) to evaluate cell viability [56–58].

#### In vitro pro-inflammatory test of LNP

To test inflammation in HaCaT cells treated with 200 µM H₂O₂ and assess the inflammation-modulating effects of Vitamin D_3_ and Vitamin D_3_-loaded nanoparticles (Vit D_3_-LNP), we first prepared a 200 µM H₂O₂ solution from a 30% w/v stock (9.8 M) [59, 60]. HaCaT cells were seeded in a 96-well plate at 1 × 10^4^ cells per well and incubated for 24 h at 37 °C with 5% CO₂. After 24 h, cells were washed with PBS and treated with 200 µM H₂O₂ for 2 h to induce inflammation, then washed with PBS again before adding Vit D_3_ or Vit D_3_-LNP, forming six experimental groups: Control, H₂O₂ only, Vit D_3_ only, Vit D_3_-LNP only, H₂O₂ + Vit D_3_, and H₂O₂ + Vit D_3_-LNP. The cells were incubated for an additional 24 h, after which a 10–20 µM DCFH-DA (DCF) working solution was prepared by dissolving DCFH-DA in PBS, diluting it in serum-free media, and adding it to the wells. Following a 30–60-min incubation in the dark, cells were washed, and ROS levels were measured using a fluorescence plate reader (excitation 485 nm, emission 535 nm). A reduction in ROS levels in the Vit D_3_ and Vit D_3_-LNP groups compared to the H₂O₂-only group indicated antioxidant effects [61, 62]. Images of these first four groups were captured using Multiphoton microscopy (MPM) after 24 h. For the last two groups (H₂O₂ + Vit D_3_ and H₂O₂ + Vit D_3_-LNP), cells were washed with PBS, treated with 200 µM H₂O₂ for 2 h, rewashed, and treated with the respective formulations (Vitamin D_3_ or Vitamin D_3_-LNP), and incubated for another 24 h. MPM images were acquired 48 h post-seeding for all six experimental groups and analyzed using SPCImage software (Becker & Hickl, Berlin, Germany) to assess morphological alterations and the effects on inflammation modulation [54, 63].

#### Statistical analysis

Statistical analyses were performed using GraphPad Prism version 10.4.1. Data are presented as mean ± SEM from triplicate experiments. The Student’s t-test was applied to determine statistical differences among the experimental groups, with a *p*-value < 0.05 considered statistically significant.

## Results and Discussion

### Quantify Vitamin D_3_

**Fig. 2:**
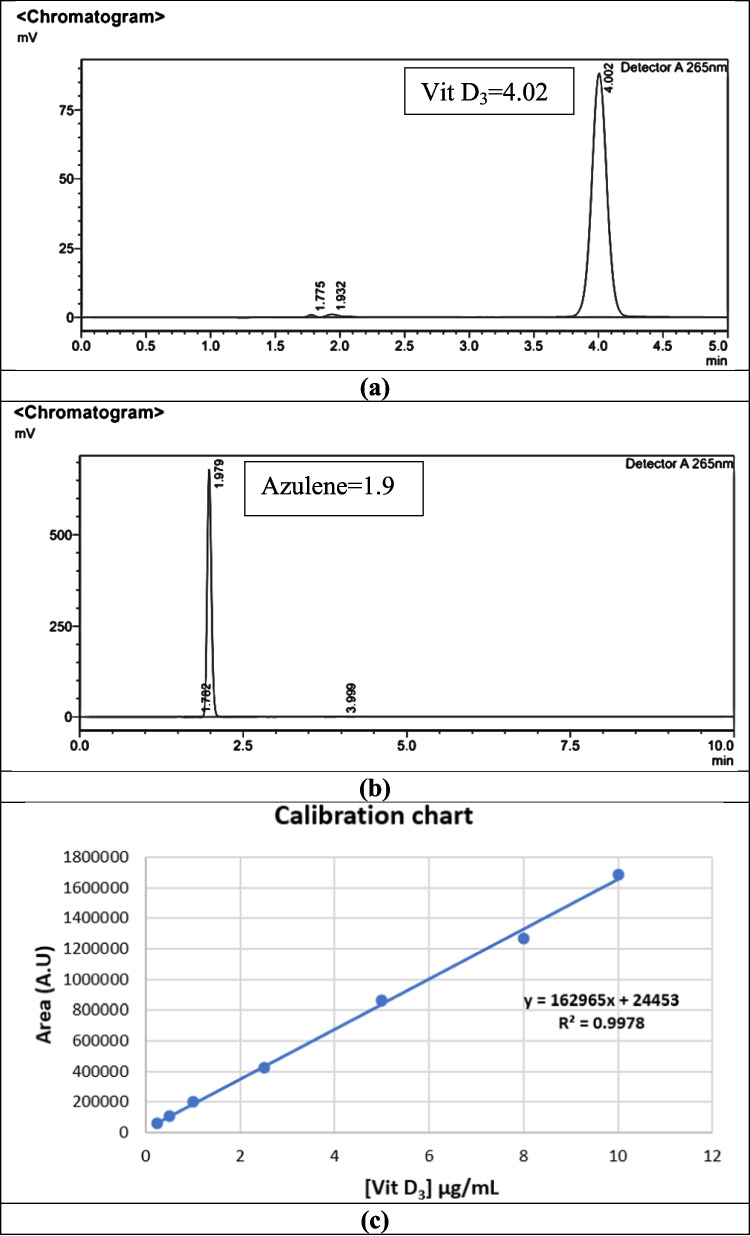
Representative HPLC chromatograms and calibration curve (**a**) Chromatogram of Vitamin D_3_ with a retention time of 4.02 min. (**b**) Chromatogram of Azulene with a retention time of 1.9 min. Both compounds were effectively separated using a validated method with a shared mobile phase. (**c**) Calibration curve of Vitamin D_3_ demonstrating a linear relationship between concentration (0.25–10 µg/mL) and peak area, with a correlation coefficient (R^2^) of 0.998

The High-Performance Liquid Chromatography (HPLC) method employed in this study successfully separated and quantified Vitamin D_3_. The experimental conditions for the chromatographic analysis were adopted from existing literature [20]. The same mobile phase experimental conditions were applied for the analysis of both Vitamin D_3_ and Azulene. The retention time for Vitamin D_3_ was determined to be 4.021 min under the specified chromatographic conditions (Fig. 2a), while Azulene was determined to be 1.979 (Fig. 2b). The two figures show sharp chromatogram peaks that did not overlap significantly with the main drug peaks.

To establish the linearity in a calibration curve, a series of Vitamin D_3_ working standard solutions, ranging from 0.25 µg/mL to 10 µg/mL, was prepared through serial dilution. The calibration curve was established by plotting the peak area against the concentration of each standard solution. The linearity of the calibration curve was confirmed, demonstrating a reliable relationship between the concentration of Vitamin D_3_ and its corresponding peak area (R^2^ = 0.998) (Fig. 2c).

### Vitamin D_3_ saturated solubility in liquid lipids

Vitamin D_3_ demonstrated negligible solubility in water (1.03 × 10⁻⁶ mg/mL at room temperature), classifying it as practically insoluble [64]. This poor aqueous solubility supports the rationale for encapsulating Vitamin D_3_ within lipid-based carriers to enhance its solubility and bioavailability. To identify suitable lipid excipients, the saturated solubility of Vitamin D_3_ was assessed in various liquid lipids, including PEG-35 Castor Oil (CO), Flaxseed Oil (FSO), Propylene Glycol Monolaurate (Lauroglycol FCC) (PML), and Medium Chain Triglycerides Caprylic/Capric Triglyceride (CCT). The testing was conducted at room temperature to avoid the need for lipid melting, considering the instability of Vitamin D_3_.

The experimental approach involved the addition of an excess amount of Vitamin D_3_ (3 mg) to 1 mL of each liquid lipid (CO and FSO), and 7 mg for each (CCT and PML) until apparent total dissolution was achieved. CO and FSO were not injected into the HPLC due to their high viscosity, which could potentially block the HPLC column. Therefore, their quantification via HPLC was not feasible in this study. CCT and PML demonstrated the ability to solubilize Vitamin D_3_ without affecting its retention time. The HPLC analysis revealed that Vitamin D_3_ was retained at 3.9 min for both CCT and PML. While CO and FSO were excluded from HPLC analysis due to concerns about device viscosity, CCT and PML were found to effectively solubilize Vitamin D_3_ without impacting its retention time. Utilizing the quantification equation derived from the Vitamin D_3_ calibration curve and considering the area under the curve for each liquid lipid and Vitamin D_3_, the concentration of Vitamin D_3_ was determined. The calculated values are expressed as Y in the equation Y = 162965X + 24,453. The Vitamin D_3_ areas under the curves are Y = 484,187 for PML and Y = 817,110 for CCT.

- For PML: 484,187 = 162965X + 24,453, resulting in X≈2.82 µg/mL.
- For CCT: 817,110 = 162965X + 24,453, resulting in X≈4.86 µg/mL.

After considering the dilution factor (200x), the solubility of Vitamin D_3_ in 1 mL of CCT is 972 µg/mL, whereas in 1 mL of PML, it is 564 µg/mL. These findings indicate a higher solubilization of Vitamin D_3_ in CCT compared to PML. Additionally, peaks on the chromatogram correspond to the concentration of Vitamin D_3_, and the area under the peaks can be used for quantitative comparisons.

### Compatibility test of solid and liquid lipids

**Fig. 3:**
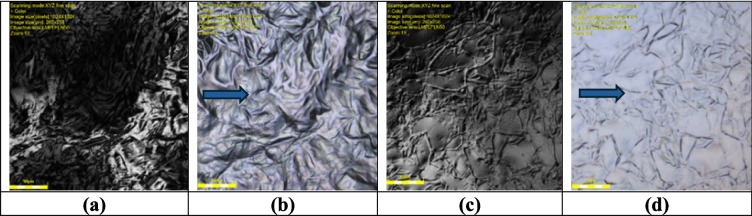
Optical microscopy images showing the compatibility study between Glyceryl Monostearate (GMS) and Tefose^®^ 1500 (PEG). (**a** and **b**) Solid lipids Glyceryl Monostearate (Kolliwax^®^ GMS II) (GMS) and (**c** and **d**) Tefose^®^ 1500 (PRG-6 Stearate/PEG-32 Stearate) (PEG) images under the optical microscopy

**Fig. 4:**
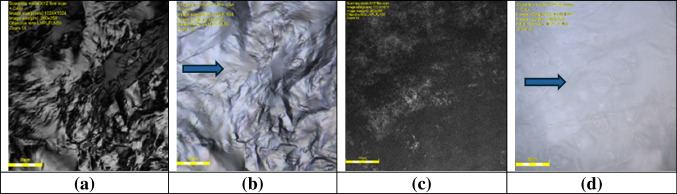
Optical microscopy images of lipid mixtures. (**a** and **b**) Glyceryl Monostearate and Caprylic/Capric Triglyceride (GMS-CCT) mixture, which shows a clear, homogeneous dispersion with minimal crystallization, indicating strong compatibility. In contrast, (**c** and **d**) Tefose^®^ 1500 and Caprylic/Capric Triglyceride (PEG-CCT) mixture exhibits visible oil droplets and delayed solidification, as marked by arrows, suggesting lower compatibility at this ratio. The images illustrate the miscibility and physical compatibility of solid and liquid lipids at a 1:1 ratio, The images were taken shortly after cooling from just above the solid lipid’s melting point

The compatibility study between solid lipids Glyceryl Monostearate (Kolliwax^®^ GMS II) and Tefose^®^ 1500 (PRG-6 Stearate/PEG-32 Stearate) (GMS and PEG), respectively, and liquid lipid Medium Chain Triglycerides Caprylic/Capric Triglyceride (CCT) was conducted using a Laser Confocal Microscope Olympus LEXT OLS5100. The intended formulation ratio of 1:1 was employed for the lipid mixture (Fig. 3). Upon slight heating above the solid lipid’s melting point, the observations revealed that both GMS and PEG exhibited compatibility with CCT (Fig. 4).

**Fig. 5:**
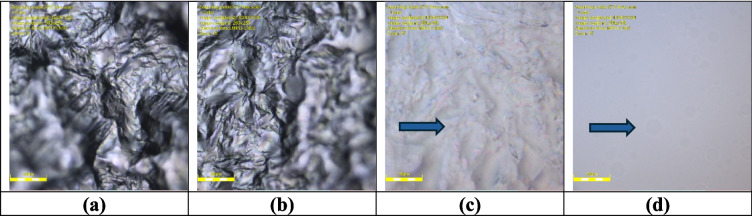
Optical microscopy images showing different ratios of lipid mixtures. (**a**) CCT-GMS at a 3:7 ratio, (**b**) CCT-GMS at a 7:3 ratio, (**c**) CCT-PEG at a 3:7 ratio, and (**d**) CCT-PEG at a 7:3 ratio. The images were captured to evaluate the effect of varying solid-to-liquid lipid ratios on miscibility and physical homogeneity. The GMS-based mixtures (**a**, **b**) display more uniform structures and better dispersion, while the PEG-based mixtures (**c**, **d**) exhibit visible oil droplets and phase separation, particularly at higher CCT content. Arrows indicate regions of phase separation or oil droplet formation in PEG-containing mixtures. Among the ratios tested, the 1:1 GMS-CCT mixture (see Fig. 4(**a**, **b**)) exhibited the highest degree of compatibility, suggesting it as the most suitable composition for subsequent formulation development

The microscopic analysis, conducted under both transmitted and polarized light, provided valuable insights into the compatibility of the lipid mixtures. The clear, homogenous appearance in both the GMS-CCT and PEG-CCT lipid mixture, as well as softer consistency and reduced crystallization, were observed, indicating good compatibility at a 1:1 ratio. However, a slight distinction emerged between the two solid lipids when changing the ratio. Specifically, GMS and CCT demonstrated a higher level of compatibility compared to PEG and CCT, as evidenced by the presence of visible oil drops in the PEG-CCT lipid mixture. In addition, PEG did not solidify at room temperature when mixed with CCT immediately; however, it took approximately 15 min to solidify *(*Fig. 5). This highlighted the potential influence of the lipid ratio on the physical characteristics of the mixture. These findings underscore the importance of lipid compatibility in pharmaceutical and cosmetic formulations, with GMS emerging as a more suitable solid lipid partner when combined with liquid lipid (CCT). Further optimization of lipid ratios may offer opportunities for modifying the properties of lipid-based formulations to meet specific application requirements.

### Formulating a lipid nanoparticle dispersion: size and zeta potential evaluation

**Fig. 6:**
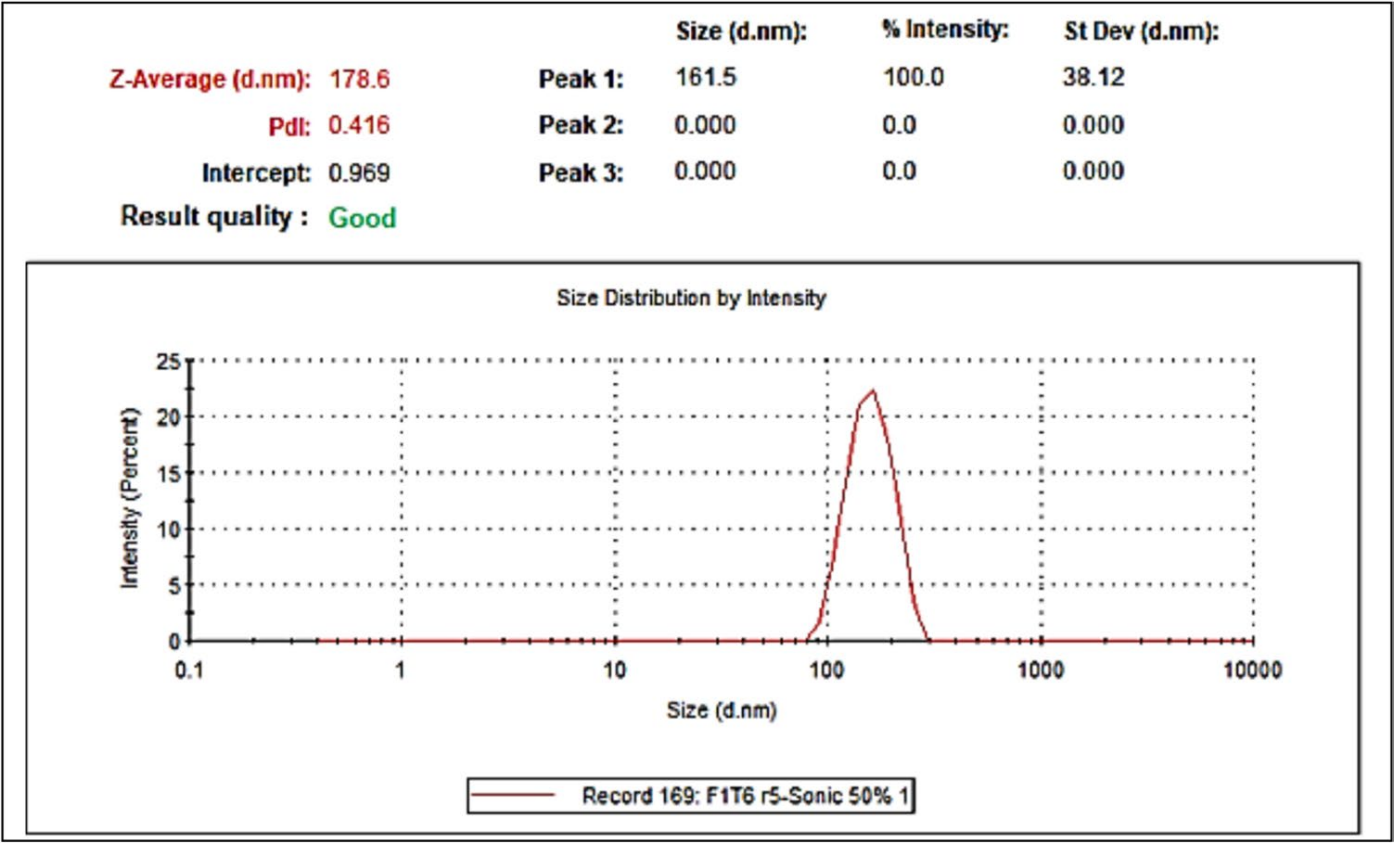
The illustration demonstrates the size and PDI of the placebo LNP formulation

The development of the placebo lipid nanoparticle dispersion utilizing the Hot Homogenization and Ultrasonication method yielded a monomodal distribution, as evidenced by a single peak in the particle size distribution analysis (Fig. 6). The initial formulation, composed of 5% Ethoxylated Oleyl Alcohol 20 OE (EOA – Chemonic OE20), 2% Medium Chain Triglycerides (Caprylic/Capric Triglyceride), 2% Glyceryl Monostearate (GMS – Kolliwax GMS II), and 50% Polyvinyl Pyrrolidone k30 aqueous dispersion (PVPk30 – Kollidon 30), demonstrated promising results with favorable collaborative assessments. The emulsification process, involving controlled heating and gradual addition of the aqueous phase into the oil phase, was critical in achieving a stable dispersion.

To impart a charge to the formulation for enhanced stability, Sodium Lauryl Sulfate (SLS), an anionic surfactant, was introduced in a ratio of (1:10) from the non-ionic surfactant. The next phase of optimization will focus on refining both the process parameters and the formulation to further enhance the nanoparticle dispersions’characteristics and performance for potential applications in drug delivery systems.

#### Process development for lipid nanoparticles

Experimental Design and Analysis: The DoE data were analyzed using Minitab software with a Box-Behnken Design, resulting in 15 experiments, each performed in triplicate. Stability was observed to decrease after 7 days, so only data from days 0 and 1 were considered. Observations at Day 0 are that the particle size was excessively small, which may be due to an excess of surfactant leading to the formation of micelles rather than lipid nanoparticles [43, 65].

Analysis of 1-Day Data: Response Surface Regression (RSR) was performed on the 1-day data. The Analysis of Variance indicated that the model as a whole was statistically significant (p < 0.001) for all parameters: z-average, PDI, and Zeta Potential. Factors such as stirring, ultrasound time, and ultrasound amplitude showed significant linear, quadratic, and interaction effects. Therefore, the RSR was used to optimize the parameters for 1-day data. Targets were set for Zeta Potential (−40 mV), PDI (0.3), and Z-average (200 nm). The software identified a solution with desirability of 97.3%, recommending stirring at 752 rpm, an ultrasound time of 15 min, and an ultrasound amplitude of 44%.

#### Formulation development for lipid nanoparticles

**Fig. 7:**
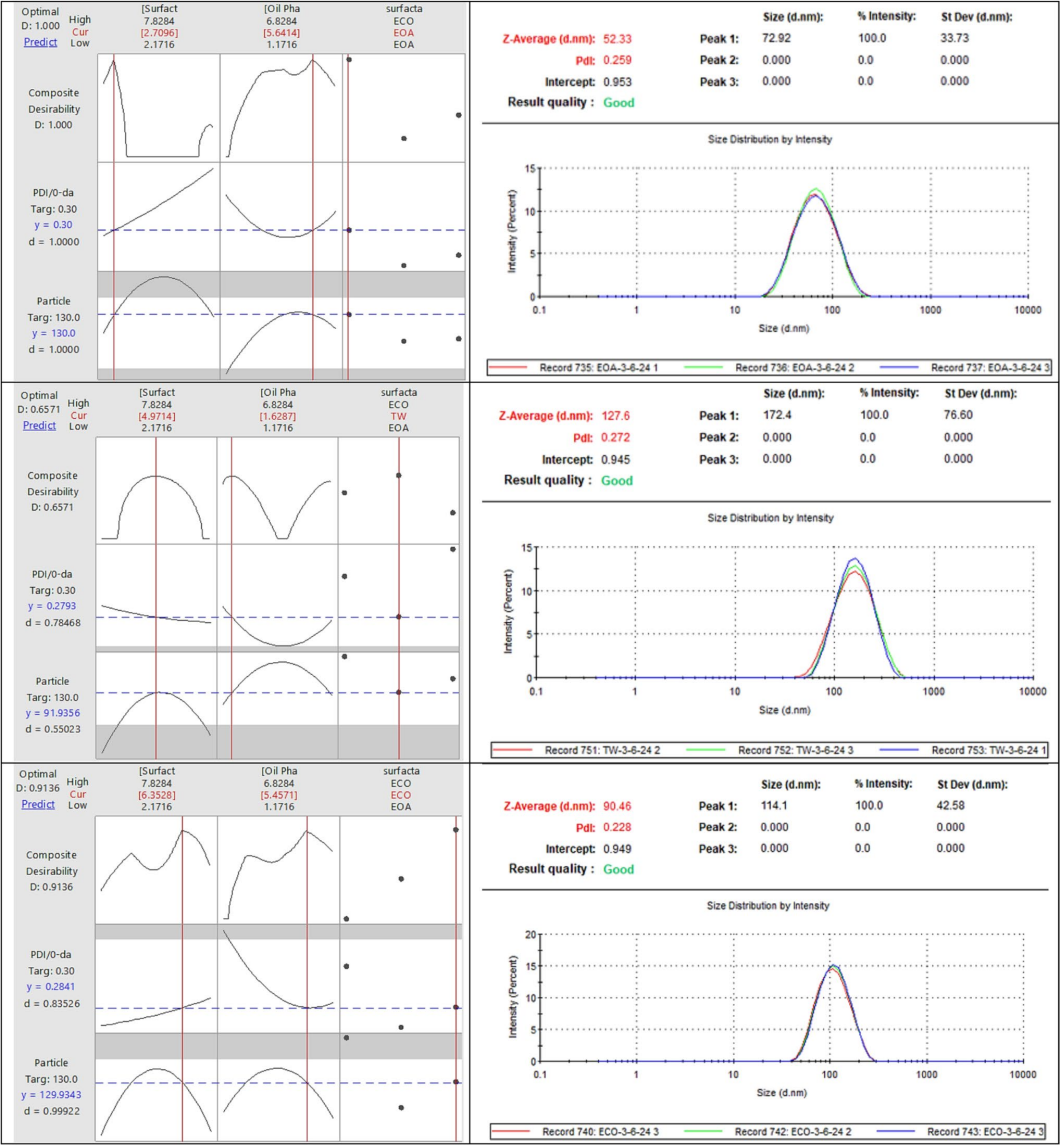
The illustration demonstrates the size and PDI of optimized LNP formulations

The goal was to target specific ranges for PDI and particle size at 0 days. The response optimization parameters were set with a PDI target range between 0.2037 and 0.474 and a particle size range between 45.37 nm and 157.533 nm, each with a weight and importance of 1. It is important to note that this range reflects the broader design space used during optimization; however, formulations with PDI values above 0.3 were not considered optimal. For the surfactant EOA, the optimal solution was found at a surfactant concentration of 2.71%w/w and an oil phase concentration of 5.64%w/w, yielding a PDI of 0.300 and a particle size of 130.0 nm with a composite desirability of 1.000. Another significant solution for the surfactant TW, with a surfactant concentration of 4.97%w/w and an oil phase concentration of 1.63%w/w, achieved a PDI of 0.2793 and a particle size of 91.9 nm with a composite desirability of 0.6571. For the surfactant ECO, the optimal parameters were a surfactant concentration of 6.35%w/w and an oil phase concentration of 5.45%w/w, resulting in a PDI of 0.2841 and a particle size of 129.934 nm with a composite desirability of 0.9136. These formulations were recommended for further testing, noting that these values are valid only at t = 0 days, as most formulations exhibited an increase in particle size over time, indicating potential destabilization. However, this trend was observed during the optimization phase; once the final optimized formulation was identified, it demonstrated stable particle size and PDI over 30 days. The multiple response predictions for these optimal settings provided 95% confidence intervals for PDI and particle size, ensuring robust evaluation and proof for development (Fig. 7).

Size and polydispersity index (PDI) of optimized lipid nanoparticle (LNP) formulations prepared with different surfactants: EOA, TW, and ECO. The figure illustrates the particle size and PDI at day 0 for each optimized formulation, demonstrating compliance with the target design space. These results supported the selection of stable and effective formulations for further development and long-term evaluation.

Imran et al., Nasiri et al., and Stefanov & Andonova have demonstrated the critical role of systematic optimization in improving nanoparticle performance. For instance, the work on α-tocopherol-loaded solid lipid nanoparticles emphasized the importance of formulation variables, such as lipid concentration and surfactant composition, in achieving desirable physicochemical characteristics [22, 66, 67]. Similarly, our study employed a design of experiments (DoE) approach to optimize surfactant and oil phase concentrations, leading to enhanced particle stability, reduced PDI, and improved encapsulation efficiency.

### Cream formulation vehicle for the lipid nanoparticles

The formulation demonstrated stability for more than one year, making it suitable to serve as a vehicle for the dispersion of Vitamin-D_3_ nanoparticles and azulene [68–72]. To this formulation, Vitamin D_3_ (cholecalciferol) was incorporated at a concentration of 0.01% w/w (1 mg per 100 g of cream), based on evidence demonstrating its topical efficacy in stimulating melanocyte activity, which is crucial for repigmentation in the treatment of vitiligo. A study using 100 µg topical Vitamin D_3_ showed increased melanocyte DOPA-positivity, supporting its biological activity at low doses [73]. Additionally, clinical findings with Topical-vitamin D gel delivering 125 µg/g Vitamin D_3_ confirmed the safety and effectiveness of the transdermal route for systemic and local delivery [74]. Azulene was incorporated at 0.03% w/w (0.3 mg/g cream), based on its demonstrated anti-inflammatory properties and its ability to reduce UVB-induced erythema comparably to a 0.5% hydrocortisone cream [2].

### Characterization of LNP loaded with Vitamin D_3_

#### Physical characterization

**Fig. 8:**
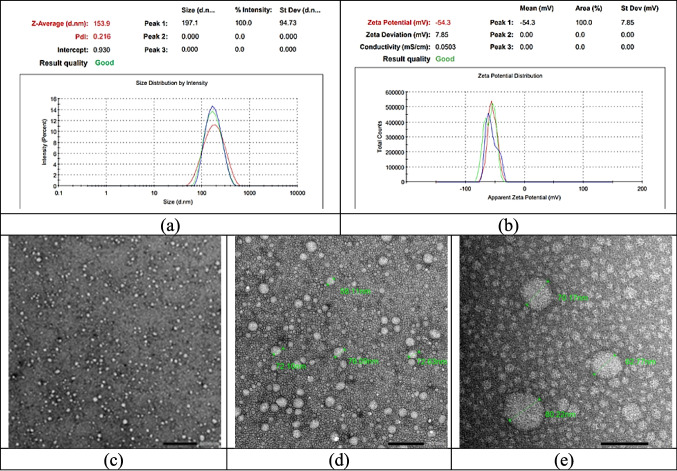
Characterization of Lipid Nanoparticles (LNP) loaded with Vitamin D_3_. (**a**) Dynamic Light Scattering (DLS) analysis showing particle size distribution and polydispersity index (PDI) of the Vit D_3_-LNP formulation, (**b**) Zeta potential distribution measured by DLS, (**c**) Transmission Electron Microscopy (TEM) image of LNP morphology at 12,000 × magnification, (**d**) TEM image at 30,000 × magnification, and (**e**) TEM image at 80,000 × magnification, highlighting nanoparticle structure and distribution

The DLS analysis provided key information on the LNPs’ hydrodynamic diameter and size distribution, with PDI values indicating the uniformity of the formulation. A PDI value below 0.3 is generally considered ideal for lipid nanoparticles, as it reflects a narrow size distribution and correlates with enhanced physical stability [19]. Zeta potential values greater than + 30 mV or less than −30 mV suggest sufficient electrostatic repulsion to prevent aggregation. Furthermore, the inclusion of non-ionic surfactants with high ethylene oxide content contributes to steric stabilization. These mechanisms collectively help to reduce flocculation, coalescence, and Ostwald ripening, thereby supporting the long-term stability of the dispersion [75, 76]. Based on the optimization results and subsequent stability testing, the formulation with surfactant ECO at a concentration of 6.35% and an oil phase concentration of 5.46% was recommended for further development. This formulation not only meets the initial target criteria for PDI and particle size but also demonstrates enhanced stability over time, making it a promising candidate for long-term applications [30]. When Vitamin D_3_ was incorporated into the ECO surfactant formulation, the particle size was observed to be 153.9 nm, with a PDI of 0.216 (Fig. 8a), which TEM confirmed (Fig. 8c,d, and e), and the zeta potential was measured as −54.3 mV (Fig. 8b).

Our formulation achieved a particle size below 200 nm with high encapsulation efficiency, consistent with trends reported in the literature. For example, Ramezanli et al. study on polymeric nanospheres for Vitamin D_3_ topical delivery emphasized the need for nanoscale carriers to improve skin penetration and loading efficiency [77]. Furthermore, Hussain, Xu et al. research on nano-based strategies for Vitamin D delivery supports the notion that reducing particle size enhances bioavailability and cellular uptake [78], which is in agreement with the design and performance outcomes of our optimized lipid nanoparticle system.

#### Thermogravimetric characterisation of Vitamin D_3_ in LNP 

**Fig. 9:**
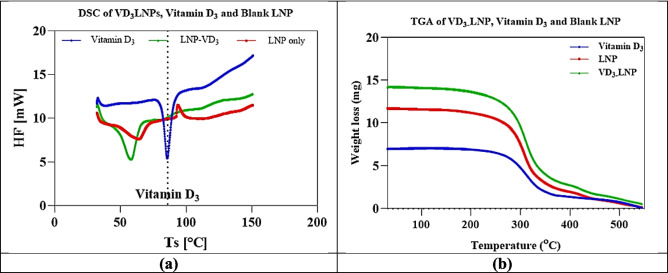
(**a**) Differential Scanning Calorimetry for Vitamin D_3_, LNP, and for Vit-D_3_-LNP, (**b**) Thermogravimetric (TGA) of Vitamin D_3_, LNP, and Vit-D_3_-LNP. (**a**) Differential Scanning Calorimetry (DSC) thermograms of pure Vitamin D_3_, lipid nanoparticles (LNP), and Vitamin D_3_-loaded lipid nanoparticles (Vit-D_3_-LNP). The DSC analysis reveal a sharp endothermic peak for pure Vitamin D_3_ at 82–86 °C (corresponding to the Vit-D_3_ melting point), indicating its crystalline nature, while the absence or broadening of this peak in Vit-D_3_-LNP confirm successful encapsulation and interaction with formulation components, including Caprylic/Capric Triglyceride (CCT), Glyceryl Monostearate (GMS – Kolliwax^®^ GMS II), and Polyvinylpyrrolidone K30 (PVP K30 – Kollidon^®^ 30). (**b**) Thermogravimetric Analysis (TGA) of pure Vitamin D_3_, LNP, and Vit-D_3_-LNP. TGA results demonstrate enhanced thermal stability of the formulations, with LNP and Vit-D_3_-LNP exhibiting a more gradual degradation profile compared to pure Vitamin D_3_. These findings confirm the successful integration of Vitamin D_3_ into the lipid-PVP matrix, improving its stability and encapsulation efficiency for potential topical delivery

DSC analysis of dried samples (5–10 mg) was performed under nitrogen, the ramp rate ranged from 30 °C to 600 °C at 10°C/min to assess the thermal behaviour of pure Vitamin D_3_ (Vit-D_3_), Vitamin D_3_-loaded lipid nanoparticles (Vit D_3_-LNP), and lipid nanoparticles (LNP). The DSC thermogram (Fig. 9a) showed a sharp endothermic peak for pure Vitamin D_3_ around 82–86°C, indicating its crystalline nature and characteristic melting behavior. However, in Vit D_3_-LNP, this peak was shifted, broadened, or diminished, suggesting encapsulation and interactions with formulation components such as PVP, GMS, and CCT. For LNP, the characteristic GMS melting peak (55–65°C) was observed. Still, it appeared broadened and slightly shifted, likely due to interactions with liquid CCT, which altered the crystalline structure and increased lipid matrix fluidity. The absence of a distinct Vitamin D_3_ peak in LNP confirmed successful encapsulation, indicating that Vitamin D_3_ was well-integrated into the lipid matrix rather than existing as a separate crystalline phase.

The Thermogravimetric Analysis (TGA) (Fig. 9b) further supported these findings by demonstrating the thermal behaviour of the formulations. Pure Vitamin D_3_ exhibited significant weight loss at lower temperatures, while both LNP and Vit D_3_-LNP showed a more gradual degradation pattern, indicating improved thermal characteristics. The presence of PVP and lipids (GMS, CCT) in the LNP system likely contributed to this effect by promoting an intimate interaction between the components, which may have restricted molecular mobility and slowed down the degradation process. While amorphization can sometimes lead to decreased stability, in this case, the positive interactions between the PVP, lipids, and Vitamin D_3_ appeared to enhance the formulation’s thermal behavior [79]. These results confirmed that Vitamin D_3_ was effectively incorporated into the lipid-polymer matrix, supporting its potential for topical delivery at physiological temperatures (32°C–37°C).

#### Determination of encapsulation efficiency

The drug-loaded lipid nanoparticles (DLPL) encapsulation efficiency assessment was conducted to determine the amount of Vitamin D_3_ successfully encapsulated within the LNP. Using high-performance liquid chromatography (HPLC) to quantify the encapsulated Vitamin D_3_, the total amount of Vitamin D_3_ in the LNP (*n* = 3) was 5.345 µg/mL. In contrast, free Vitamin D_3_ (*n* = 3) was 0.161 µg/mL.

The encapsulation efficiency was 96.98%, indicating a high level of Vitamin D_3_ encapsulation within the lipid nanoparticles.

To validate the method, a mass balance analysis was also performed by summing the encapsulated and free drug quantities and comparing them with the initial amount added. The mass balance recovery was 103.08%, confirming the accuracy and reliability of the encapsulation efficiency determination.

#### Chemical stability of Vitamin D_3_ loaded in LNP

**Fig. 10:**
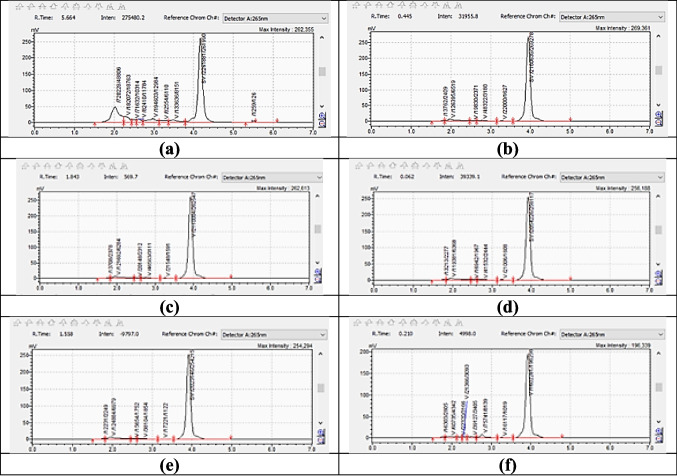
HPLC Analysis Results for Vitamin D_3_ Stability Under Different Storage Conditions after 30 Days. (**a**) Vitamin D_3_ reference concentration (initially 10 µg/mL). (**b**) Vitamin D_3_ degradation in LNPs stored in the dark: minimal loss (6%), confirming the protective effect against light exposure. (**c**) Under continuous light exposure, degradation increased to 9%, demonstrating Vitamin D_3_’s photosensitivity and supporting the need for dark storage to avoid light-induced isomer formation. (**d**) Refrigerated storage at 4 °C resulted in a 13% reduction, indicating moderate improvement in stability. (**e**) Freezing at –20 °C resulted in a 15% reduction, indicating only a slight enhancement over refrigeration. (**f**) Elevated temperature storage at 40 °C caused the most significant degradation (40%), highlighting the temperature sensitivity of Vitamin D_3_ and the importance of avoiding heat during storage

The HPLC analysis revealed the percentage reductions in Vitamin D_3_ concentration (starting at 10 µg/mL) in lipid nanoparticles (LNP) under different storage conditions after 30 days (Fig. 10). At room temperature, samples stored in the dark storage showed a 6% reduction in Vitamin D_3_ content, confirming the protective effect against light exposure. In contrast, room temperature storage under continuous light exposure resulted in a 9% reduction, highlighting Vitamin D_3_’s photosensitivity and the need for storage in dark environments to prevent light-induced degradation and inactive isomer formation [80, 81].

The effect of temperature was even more pronounced. Storage at 40 °C led to the highest degradation, with a 40% reduction, indicating that high temperatures drastically accelerate Vitamin D_3_ breakdown. In cool storage conditions, degradation was 13% at 4 °C and 15% at −20 °C, suggesting that while refrigeration slows degradation, cooling offers only a slight improvement in stabilization. These findings emphasize that proper storage in dark, room-temperature conditions is crucial to minimizing Vitamin D_3_ degradation and maintaining stability [80, 82, 83]. Consistent with our results, previous studies have shown that Vitamin D_3_ predominantly undergoes photochemical degradation into biologically inactive isomers, including tachysterol, lumisterol, and isotachysterol, under standard storage conditions. Notably, no pharmacologically active degradation products have been identified as a result of non-enzymatic degradation pathways of Vitamin D_3_ [82, 83]. This supports the chemical stability profile observed in our formulation, in which no additional peaks corresponding to active degradation products were detected by HPLC analysis.

### Characterization of cream loaded with LNP

**Fig. 11:**
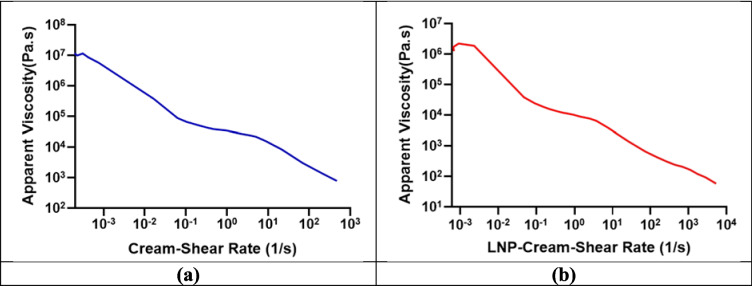
Rheological profiles of the base cream and LNP-loaded cream (LNP-Cream)at 25 °C. These profiles show shear-thinning (pseudoplastic) behavior (**a**) for the cream and (**b**) for the LNP-cream. Viscosity decreases with increasing shear rate, demonstrating reduced flow resistance under mechanical stress

The rheological analysis of both the base cream and the LNP-loaded cream (LNP-Cream) confirmed non-Newtonian, shear-thinning (pseudoplastic) behavior. As shear rate increased from very low values (~ 0.00008 s⁻1 for cream and ~ 0.00068 s⁻1 for LNP-Cream) to high shear rates (~ 5350 s⁻1 for LNP-Cream and ~ 474 s⁻1 for cream), the viscosity of both formulations decreased markedly, spanning several orders of magnitude (Fig. 11). This shear-thinning profile is characteristic of semisolid topical systems, indicating that both formulations exhibit reduced resistance to flow under mechanical stress, such as rubbing or spreading during application. This behavior supports the creams’ suitability for topical use, as they provide high viscosity at rest for stability, while allowing ease of application under shear. The yield stress of the LNP-cream was also lower than that of the base, indicating a reduced force requirement for initial flow. This property can enhance the user experience during the application. These trends are consistent with previous findings where nanoparticle inclusion modified the internal cream structure without compromising its physical stability [34, 38, 84].

**Fig. 12:**
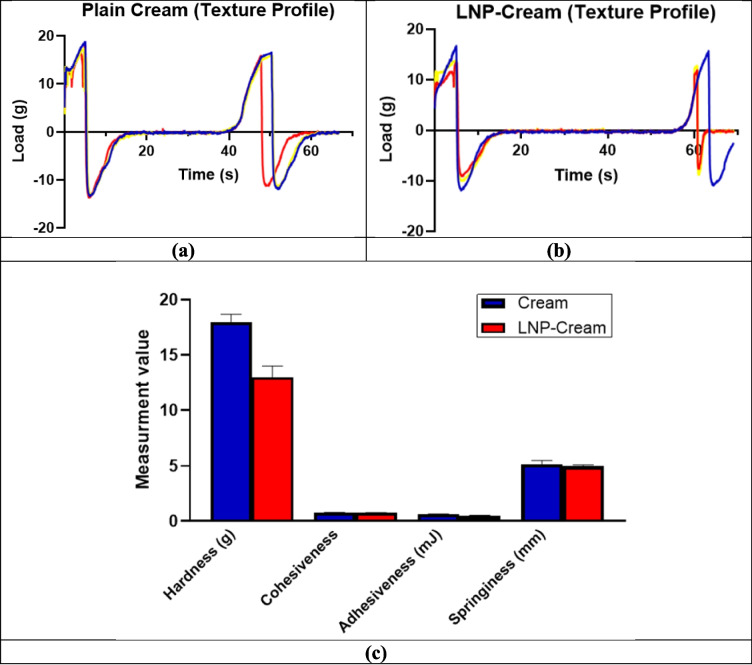
Texture profile analysis of the base cream and LNP-loaded cream (LNP-Cream). (**a**) and (**b**) show the force-distance curves obtained from double compression cycle tests. (**c**) Quantitative comparison of texture parameters including hardness, adhesiveness, cohesiveness, and springiness. The LNP-Cream exhibited significantly lower hardness (*P* < 0.05) compared to the base cream, indicating a softer texture. Adhesiveness, cohesiveness, and springiness showed slight, non-significant (NS) reductions, suggesting preserved structural integrity and elasticity

Texture profile analysis further supported this observation (Fig. 12a and b). The LNP-loaded cream exhibited a reduction in hardness (12.97 g) compared to the control cream (17.97 g) (*P* < 0.05), indicating a softer texture (Fig. 12c). Adhesiveness and cohesiveness also declined slightly in the LNP formulation, which may improve tactile perception by reducing stickiness while maintaining structural integrity. Springiness showed only a minimal decrease, indicating that the formulation retained adequate elasticity after deformation. These outcomes align with those of Dabbaghi et al., who emphasized the importance of maintaining viscoelastic properties to ensure consistent product performance and user acceptability [33].

Together, these results confirm that the incorporation of lipid nanoparticles into the topical cream vehicle did not compromise its stability or performance. On the contrary, the observed reduction in viscosity and softening of texture in the LNP-loaded cream imparted functional advantages, potentially enhancing spreadability, user comfort, and overall application performance.

### In vitro skin penetration test

**Fig. 13:**
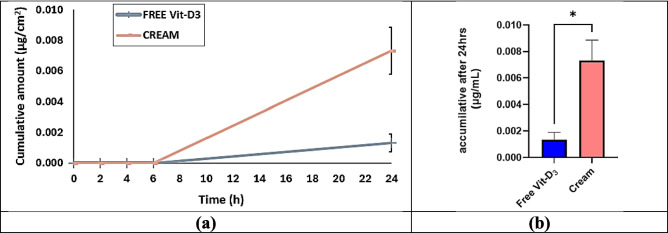
In vitro permeation test using three different donors: (**a**) and (**b**) Mean Cumulative amount (µg/cm^2^—All time points. (**a**) and (**b**) Mean cumulative drug amount (µg/cm^2^) at all time points (0, 2, 4, 6, and 24 h) following the in vitro penetration test (IVPT). The results show a significant difference (*P* < 0.05) between free Vitamin D_3_ and the cream formulation. Free Vitamin D_3_ exhibited minimal permeation, with only 0.001–0.002 µg/cm^2^ detected at 24 h, whereas the cream formulation demonstrated significantly higher permeation, reaching 0.006–0.009 µg/cm^2^. This suggests that the lipid nanoparticle (LNP) cream formulation enhances Vitamin D_3_ delivery by facilitating diffusion through the stratum corneum

The cumulative permeation results across all time points (0, 2, 4, 6, and 24 h) revealed a significant difference between the free Vitamin D_3_ and the cream formulation, *P* < 0.05 (Table 4) and (Fig. 13a and b). Free Vitamin D_3_ exhibited negligible permeation across the epidermis, with only a minimal cumulative amount being detected at 24 h (0.001–0.002 µg/cm^2^). In contrast, markedly higher permeation was recorded for the cream formulation, with cumulative amounts reaching 0.006–0.009 µg/cm^2^ at 24 h. These findings were interpreted to highlight the ability of the cream formulation to enhance skin permeation and delivery compared to free Vitamin D_3_, likely due to the lipid nanoparticle (LNP) carrier system and the cream base’s capacity to facilitate Vitamin D_3_’s diffusion through the stratum corneum.

**Table 4:** In vitro permeation test using three different donors: Mean Cumulative amount (µg/cm^2^)—All time points. Mean cumulative amount of Vitamin D_3_ permeated (µg/cm^2^) at each time point (0, 2, 4, 6, and 24 h) following in vitro skin permeation test (IVPT) using three different donors. Values are presented as mean and standard deviation (SD)

Time (h)	FREE Vit-D_3_	CREAM	SD-Vit-D_3_	SD-CREAM
0	0.000	0.000	0.000	0.000
2.00	0.000	0.000	0.000	0.000
4.00	0.000	0.000	0.000	0.000
6.00	0.000	0.000	0.000	0.000
24.00	0.001	0.007	0.0006	0.0015

**Fig. 14:**
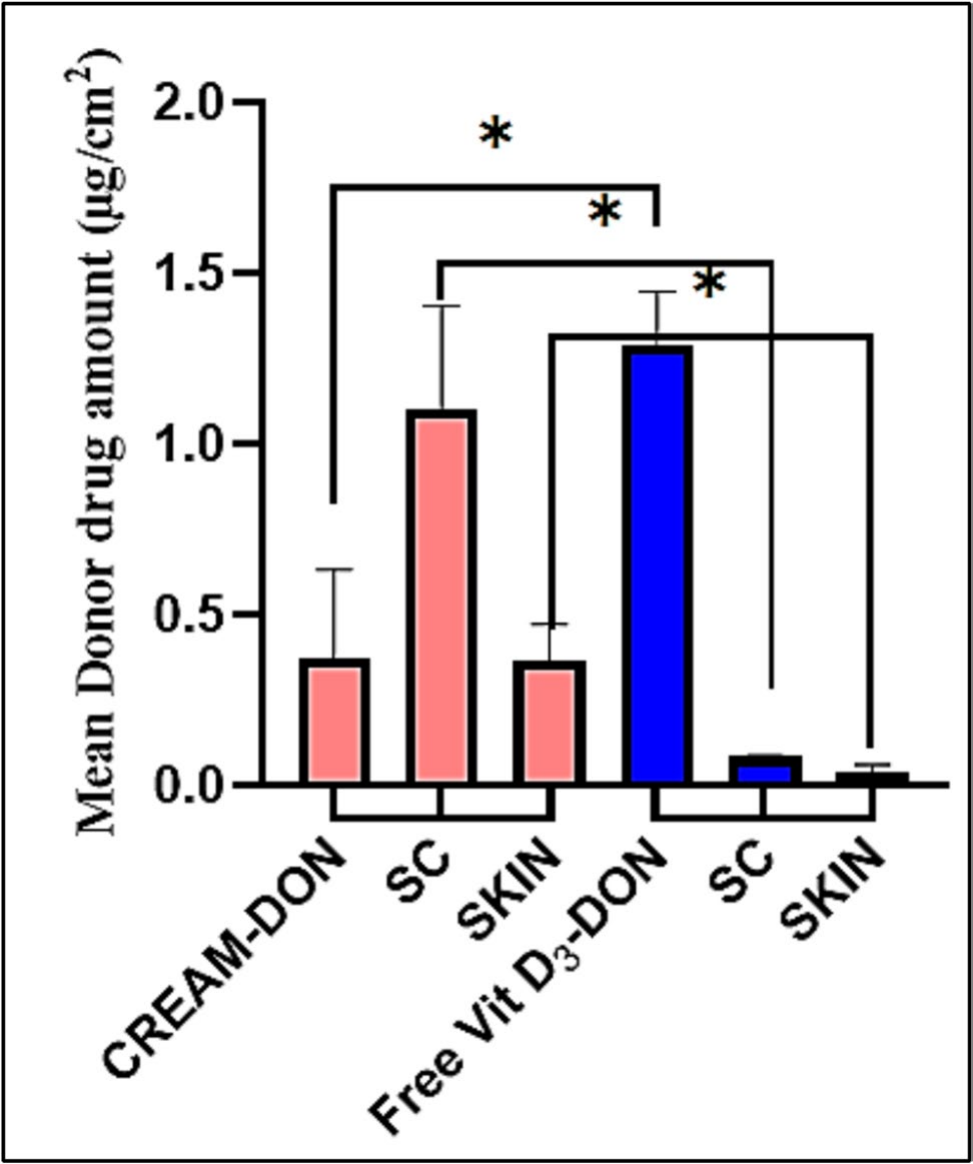
In vitro permeation test using three different donors. Mean Donor Drug Amount (µg/cm.^2^). Mean Donor drug amount (µg/cm2) following the in vitro penetration test (IVPT). The results indicate a significantly higher residual drug amount in the donor phase for free Vitamin D_3_ (1.1–1.38 µg/cm^2^) compared to the cream formulation (0.18–0.67 µg/cm^2^) (*P* < 0.05). This suggests improved skin permeation and retention of Vitamin D_3_ when incorporated into the lipid nanoparticle (LNP) cream formulation

Similarly, differences in drug retention in the donor phase, stratum corneum (SC), and deeper skin layers were indicated by the results (Table 5). Free Vitamin D_3_, high donor phase drug amounts (1.1–1.38 µg/cm2), and very low SC and skin content (0.006–0.085 µg/cm2) confirmed limited diffusion and retention. Conversely, the cream formulation exhibited lower residual drug in the donor phase (0.18–0.67 µg/cm2) (*P* < 0.05), significantly higher SC content (0.76–1.35 µg/cm2) (*P* < 0.05), and superior drug retention in deeper skin layers (0.25–0.47 µg/cm2) (*P* < 0.05) (Fig. 14) suggesting its enhanced delivery potential.

**Table 5:** In vitro permeation test using three different donors. Mean Donor Drug Amount (µg/cm^2^). Vitamin D_3_ distribution across the donor phase, stratum corneum (SC), and deeper skin layers after 24 h of in vitro permeation using three different donors. Results are expressed as mean drug amount (µg/cm^2^) and standard deviation (SD)

CREAM			
		**Mean**	**SD**
Donor-1	DON	0.667	0.176
	SC	0.764	0.076
	SKIN	0.468	0.226
Donor-2	DON	0.279	0.1
	SC	1.184	0.078
	SKIN	0.255	0.053
Donor-3	DON	0.178	0.053
	SC	1.354	0.064
	SKIN	0.375	0.027
**Free Vitamin D**_**3**_			
		**Mean**	**SD**
Donor-1	DON	1.373	0.064
	SC	0.081	0.015
	SKIN	0.045	0.046
Donor-2	DON	1.108	0.015
	SC	0.088	0.015
	SKIN	0.006	0.003
Donor-3	DON	1.386	0.067
	SC	0.085	0.031
	SKIN	0.055	0.029

These findings confirmed that the cream formulation promotes higher drug retention in the SC and deeper skin layers, which is crucial for localized therapeutic effects. The observed differences can be attributed to the lipid nanoparticle system’s ability to enhance the solubility, stability, and partitioning of Vitamin D_3_ into the skin, as well as reduce aggregation and improve dispersibility in the cream formulation compared to the free form of Vitamin D_3_. The mass balance values further validated the experimental setup, with mean recoveries of 65.77% for free Vitamin D_3_ and 81.27% for the cream formulation (Table 6). (The high P-value is likely due to the variation between the three different skin types). The lower recovery of free Vitamin D_3_ reflected its poor skin permeation and potential degradation. In contrast, the cream formulation’s higher recovery underscored its superior stability and enhanced retention in skin layers, likely due to LNP stabilization. These results align with previous studies, which demonstrate that lipid-based formulations achieve better permeation, stability, and retention of poorly soluble compounds compared to free drug solutions. Cream formulations often provide additional benefits through their excipients, hydrating agents, and vehicle properties [85–87].

**Table 6:** In vitro permeation test using three different donors, Mean Mass Balance (%)

Vitamin D_3_		Cream	
Donor-1		Donor-1	
mass balance	67.65	mass balance	66.65
Donor-2		Donor-2	
mass balance	49.56	mass balance	86.05
Donor-3		Donor-3	
mass balance	80.10	mass balance	91.10

The results of this study align with existing literature supporting lipid-based nanoparticles as promising carriers for hydrophobic bioactives, including Vitamin D_3_. Al-Smadi, Ali et al., Favas, Almeida et al., and Majeed and Rather have shown that nanostructured lipid carriers (NLCs) and solid lipid nanoparticles (SLNs) significantly improve the stability and cutaneous delivery of lipophilic molecules such as Vitamin D and marine-derived compounds [60, 88, 89]. The structural integrity, biocompatibility, and sustained release potential of LNP demonstrated in our formulation are consistent with these previously reported advantages.

### Two-photon penetration studies

**Fig. 15:**
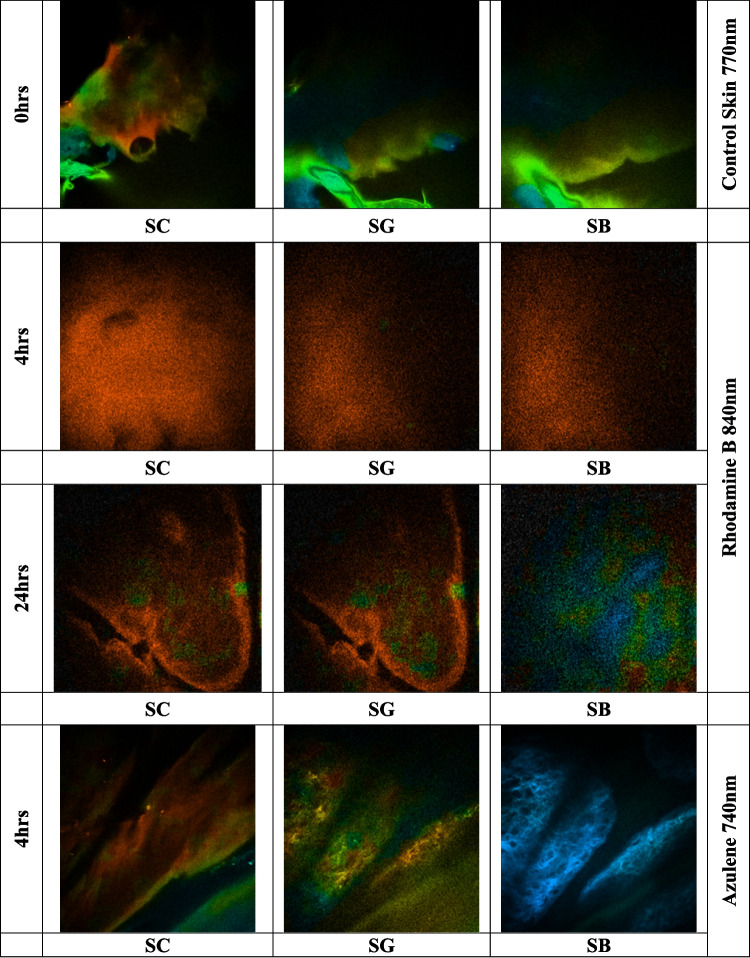

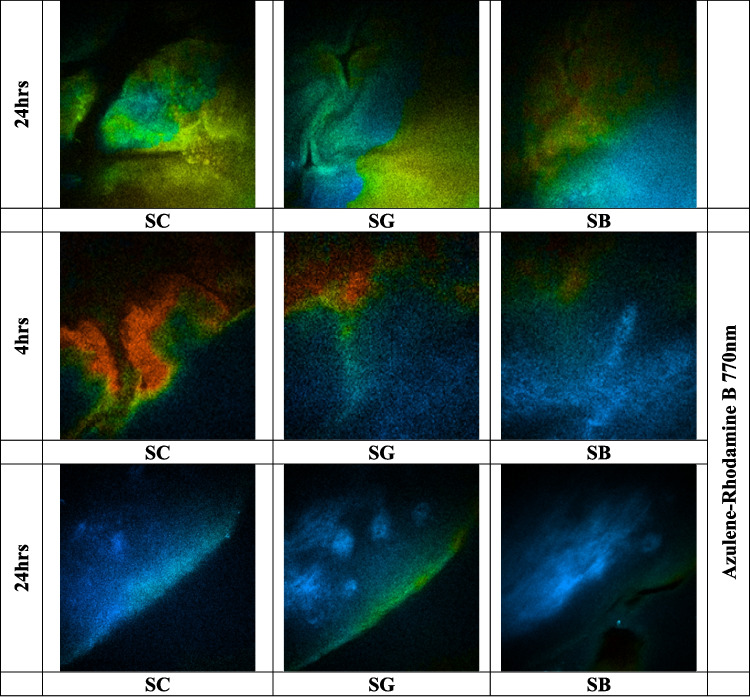
Multiphoton imaging (MPM) penetration images. Multiphoton microscopy (MPM) was used to evaluate the permeation and distribution of Rhodamine B-labelled lipid nanoparticles (LNP) and a cream containing Azulene across skin layers. Rhodamine B-labelled LNP (0.5% w/w dye) and Azulene cream were applied to frozen skin samples with a diffusion area of 1.13 cm^2^, and imaging was performed at 0, 4, and 24 h using a DermaInspect^®^ multiphoton system. Fluorescence channels: red indicates Rhodamine B-labeled LNP; blue shows skin autofluorescence. Excitation wavelengths of 740 nm, 840 nm, and 770 nm were used for autofluorescence, Rhodamine B, and the combined formulations, respectively. In comparison, **FLIM** measurements captured fluorescence decay curves for Azulene (430–450 nm) and Rhodamine B (560–580 nm). Results showed that Rhodamine B-labelled LNP penetrated all skin layers at 4 h, with reduced fluorescence in deeper layers at 24 h. In contrast, Azulene cream demonstrated consistent fluorescence across all layers at both time points. **FLIM** imaging further visualized fluorescence distribution, confirming effective delivery and retention of both formulations, with Azulene dominating fluorescence in the combined system, indicating enhanced permeation and retention properties

Multiphoton microscopy (MPM) was employed to evaluate the permeation of Rhodamine B-labelled lipid nanoparticles (LNPs) and the cream vehicle containing Azulene across the skin layers. Fluorescence intensity and distribution patterns were analyzed for each formulation over incubation periods of 4 and 24 h (Fig. 15).

Control skin (before treatment) was imaged at 770 nm to confirm baseline autofluorescence. At 4 h, Rhodamine B-labelled LNP exhibited strong fluorescence signals that penetrated all the skin layers, including the stratum corneum (SC), the stratum granulosum (SG), and the stratum spinosum (SB), as evaluated at 840 nm. This result demonstrates the ability of LNP to facilitate the delivery of encapsulated compounds through the lipid-rich layers of the skin. However, at 24 h, fluorescence was still observed in the SC and SG, while the signal in the SB was significantly reduced, likely due to decreased nanoparticle presence in deeper layers over time. For the cream containing Azulene, fluorescence was consistently observed across all skin layers (SC, SG, and SB) at both 4 and 24 h. Autofluorescence imaging at a 740 nm excitation wavelength confirmed Azulene’s distribution throughout the layers. The stable fluorescence signal highlights the cream’s effectiveness in enhancing Azulene’s permeation and retention. Azulene’s strong interaction with lipid-rich environments likely facilitated its consistent presence across the skin layers, indicating its potential for topical delivery applications. The combined system of Rhodamine B-labelled LNP and Azulene cream was analyzed at an excitation wavelength of 770 nm. After 4 h, fluorescence signals from both components were observed in the SC, SG, and SB layers, with Azulene displaying stronger fluorescence. At 24 h, fluorescence persisted across all layers, with Azulene continuing to dominate.

These findings suggest that the cream vehicle plays a vital role in sustaining the release of LNP and enhancing its permeation over time. This underscores the potential of Rhodamine B-labelled LNP loaded with Vitamin D_3_ for effective topical delivery by enabling the penetration of LNP into the SC, SG, and SB within 4 h, demonstrating their ability to transport encapsulated compounds across skin barriers [3, 90, 91]. However, the decline in fluorescence signals in the SB after 24 h highlights the need to address long-term stability and retention. This further emphasizes the importance of the cream vehicle in improving release and permeation, reinforcing its potential as an effective delivery system [77, 92–94].

### In vitro pro-inflammatory model and cell viability test of LNP

**Fig. 16:**
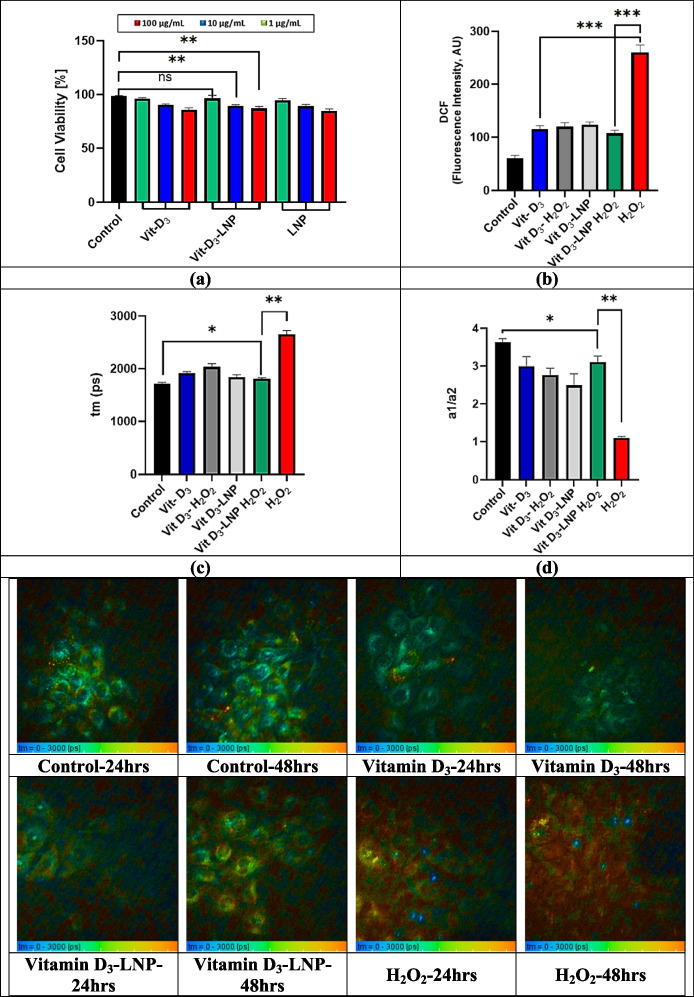

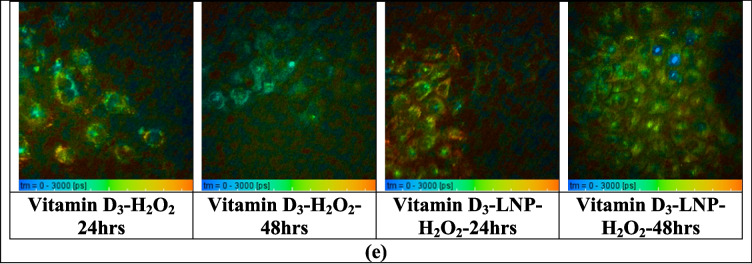
In Vitro Tests of Cell Viability, Inflammation, and multiphoton microscopy (MPM) images for LNP. (**a**) Cell Viability of free Vitamin D_3_ and Vitamin D_3_-LNP was evaluated in HaCaT cells after 24 h of exposure at concentrations of 1, 10, and 100 µg/mL. Cell viability (%) was calculated using the formula Cell Viability (%) = (Treated cells-Media)/(untreated cells-Media)*100. Both formulations maintained high cell viability (80–100%) across all concentrations, indicating no significant cytotoxic effects. (**b**) Inflammation test showing ROS levels in HaCaT cells treated with hydrogen peroxide (H₂O₂, 200 µM) with or without free Vitamin D_3_ and Vitamin D_3_-LNP. Intracellular ROS was detected using the DCFH-DA probe (DCF). The unit for DCF fluorescence intensity is typically relative fluorescence units (RFU) or simply arbitrary units (AU). H₂O₂ treatment significantly increased ROS levels (~ 250 AU), while both free Vitamin D_3_ and Vitamin D_3_-LNP significantly reduced ROS levels (***P < 0.001***), indicating strong antioxidant activity. (**c**), (**d**) and (**e**) Multiphoton Fluorescence Lifetime Imaging Microscopy (**MPM-FLIM**) images were captured after 24 h for the first four groups. Pseudocolored images are scaled from 0 to 3000 picoseconds (ps), with blue indicating shorter τm and red indicating longer τm. For the H₂O₂ + Vit D_3_ and H₂O₂ + Vit D_3_-LNP groups, cells were treated with 200 µM H₂O₂ for 2 h, followed by the respective formulations and incubation for an additional 24 h. The **FLIM** images demonstrated that Vitamin D_3_-LNP provides greater protection against H₂O₂-induced oxidative stress by restoring normal cell morphology and redox homeostasis. The H₂O₂ group showed the highest τ*m* (~ 2600 ± 71.67 ps) (red), and lowest redox ratio (1.10 ± 0.04), reflecting severe oxidative stress. Vitamin D_3_-LNP-H₂O₂ treatment significantly reduced τm (~ 1800 ± 17.23 ps) (exhibited a shift toward blue) and improved the redox ratio (3.10 ± 0.17), closer to control levels (blue) (3.63 ± 0.10), highlighting its enhanced therapeutic potential

The cell viability test demonstrated that both free Vitamin D_3_ and its lipid nanoparticle (LNP) formulation maintained high cell viability (~ 80–100%) across all tested concentrations (1, 10, and 100 µg/mL) in HaCaT cells (Fig. 16a), (note that the 100 and 10µg/mL concentrations are the concentrations of Vitamin D_3_ in the LNP and cream formulations, respectively**)**. This indicates that neither formulation exhibits significant cytotoxic effects, confirming their biocompatibility. The encapsulation of Vitamin D_3_ into lipid nanoparticles did not compromise cellular safety, supporting its potential for topical applications and extended use in skin-related treatments.

In the inflammation test, treatment with H₂O₂ resulted in a significant increase in intracellular ROS levels, as evidenced by increased DCF fluorescence intensity, accompanied by morphological changes indicative of oxidative stress (Fig. 16b). Both free Vitamin D_3_ and Vitamin D_3_-LNP treatments effectively reduced ROS levels (the *P* < 0.001 compared to the H_2_O_2_-treated group), demonstrating strong antioxidant effects. The reduction was slightly more pronounced with Vitamin D_3_-LNP, suggesting improved anti-inflammatory properties due to the nanoparticle delivery system, which likely enhances bioavailability and provides sustained release. Multiphoton microscopy (MPM) imaging supported these findings, showing improved cell morphology and reduced oxidative damage in the Vitamin D_3_ and Vitamin D_3_-LNP groups compared to the H₂O₂-only group (Fig. 16c, d, and e). MPM-FLIM images illustrate the effects of various treatments on HaCaT cells, with pseudocoloring applied according to mean fluorescence lifetime (τm) values ranging from 0 to 3000 ps. A blue-green–red color scale is used, where blue represents lower τm values and red indicates higher τm values. Among all treatments, Vitamin D_3_-LNP provided superior protection, restoring normal cellular morphology and highlighting its potential as a therapeutic option for managing inflammation-related conditions, such as vitiligo [29, 30]. τm (mean fluorescence lifetime) and a1/a2 (redox ratio) results provide insights into cells’oxidative stress and redox state under different treatments [95, 96]. The τm values for the control group, Vitamin D_3_ (Vit D_3_), Vit D_3_-H_2_O_2_, Vit D_3_-LNP, Vit D_3_-LNP-H_2_O_2_, and H_2_O_2_ treatment indicate significant alterations in the cellular microenvironment (Fig. 16c). Notably, the highest τ*m* is observed in the H_2_O_2_-treated group (~ 2600 ± 41.37 ps (picoseconds), reflecting increased oxidative stress due to ROS generation, as H_2_O_2_ is a known pro-oxidant. The Vit D_3_-H_2_O_2_ group exhibits a lower τ*m* (~ 2000 ± 36.51 ps), suggesting that Vitamin D_3_ partially mitigates oxidative stress. Interestingly, the Vit D_3_-LNP-H_2_O_2_ group shows an even lower τ*m* (~ 1800 ± 9.94 ps), the *P* < 0.01 compared to the H_2_O_2_-treated group, and *P* < 0.05 compared to the control cell, indicating that the lipid nanoparticle (LNP) formulation may provide enhanced protective effects against ROS-induced stress, potentially by stabilizing and improving the bioavailability of Vitamin D_3_.

The a1/a2 (redox ratio) further supports these observations (Fig. 16d). The control group maintains a redox ratio of (3.63 ± 0.05), which decreases significantly in the H_2_O_2_ group (1.10 ± 0.02), reflecting a shift towards free NAD(P)H and glycolytic metabolism under oxidative stress. Treatment with Vitamin D_3_ or its LNP formulation restores the redox ratio to varying extents. The Vit D_3_ group shows a ratio of (2.76 ± 0.10), indicating partial recovery, while the Vit D_3_-LNP-H_2_O_2_ group achieves a ratio of (3.10 ± 0.09), closer to control levels, the *P* < 0.01 compared to H_2_O_2_-treated group and P < 0.05 compared to control cell, These results suggest that the LNP formulation improves the delivery of Vitamin D_3_ and enhances its protective role in maintaining redox homeostasis under oxidative conditions. Together, these findings highlight the potential of Vitamin D_3_-LNP as a therapeutic strategy to combat oxidative stress and its associated cellular damage.

In line with our in vitro biocompatibility results, neither the blank lipid nanoparticles (LNP) nor the Vitamin D_3_-loaded LNP induced a significant pro-inflammatory response, underscoring the biocompatibility of the lipid carrier system. These findings are consistent with Cristelo, Sá et al. and Hussain, Xu et al. researches, which demonstrate that lipid-based nanocarriers, including Vitamin D_3_-loaded formulations, do not activate inflammatory pathways and maintain high cellular tolerance in various models, such as INS-1E cells and keratinocytes [78, 97]. In our study, both free Vitamin D_3_ and its LNP formulation preserved cell viability and did not trigger oxidative stress–related inflammatory responses in our DCF-based assay, supporting their potential for safe topical application in inflammation-associated skin conditions.

## Conclusion

This study successfully developed and optimized a Vitamin D_3_-loaded lipid nanoparticle (LNP) system integrated into an azulene cream for enhanced transdermal delivery. The findings demonstrate the potential of LNPs as an advanced topical delivery platform to improve the solubility, stability, and skin penetration of Vitamin D_3_, addressing key challenges associated with conventional cream formulations. The optimized formulation achieved high encapsulation efficiency, favorable biocompatibility, and sustained release, enabling targeted delivery to deeper skin layers and offering a promising therapeutic strategy for managing skin conditions such as vitiligo.

Future research will focus on in vivo evaluation of skin permeation, bioavailability, and therapeutic efficacy, as well as clinical studies assessing long-term patient acceptability. Additional investigations will aim to refine deposition techniques and explore alternative biodegradable lipid carriers to further enhance formulation performance and stability.

## Data Availability

The datasets generated and/or analyzed during the current study are available from the corresponding author on reasonable request.
